# CLT for NESS of a reaction-diffusion model

**DOI:** 10.1007/s00440-024-01293-1

**Published:** 2024-07-15

**Authors:** P. Gonçalves, M. Jara, R. Marinho, O. Menezes

**Affiliations:** 1 Center for Mathematical Analysis, Geometry and Dynamical Systems, Instituto Superior Técnico, Universidade de Lisboa, 1049-001 Lisbon, Portugal; 2 Instituto de Matemática Pura e Aplicada, Estrada Dona Castorina 110, Rio de Janeiro, 22460-320 Brazil; 3 Universidade Federal de Santa Maria, Campus Cachoeira do Sul, Rod. Taufik Germano, 3013, Cachoeira do Sul, 96503-205 Brazil; 4 Instituto de Matemática, UFBA, Campus de Ondina, Av. Adhemar de Barros, S/N, Salvador, CEP 40170-110 Brazil

**Keywords:** Exclusion process, Non-equilibrium state, Fluctuations, SPDEs, 60K35, 82C22

## Abstract

We study the scaling properties of the non-equilibrium stationary states (NESS) of a reaction-diffusion model. Under a suitable smallness condition, we show that the density of particles satisfies a law of large numbers with respect to the NESS, with an explicit rate of convergence, and we also show that at mesoscopic scales the NESS is well approximated by a local equilibrium (product) measure, in the total variation distance. In addition, in dimensions $d \le 3$ we show a central limit theorem for the density of particles under the NESS. The corresponding Gaussian limit can be represented as an independent sum of a white noise and a massive Gaussian free field, and in particular it presents macroscopic correlations.

## Introduction

Non-equilibrium stationary states (NESS) describe the large-time behavior of stochastic interacting systems that are kept out of equilibrium by the action of external forces. A main characteristic of NESS is the presence of *steady flows*, which can manifest themselves as energy flows, particle flows or mass flows. NESS typically appear when a closed system is kept in contact with several *reservoirs* with different thermodynamic parameters, such as temperature or chemical potential.

When stochastic interacting systems are modelled by Markov chains, the NESS have a simple probabilistic interpretation. An invariant, ergodic measure of a Markov chain is a NESS if it is not reversible with respect to the generator of the Markov chain.

In this article, we propose a general mathematical framework to describe the NESS of *driven diffusive systems*. In order to keep the technical parts at an acceptable level, we consider one of the simplest models of driven diffusive systems, the so-called *reaction-diffusion model* introduced in [9], with *quadratic* reaction term. This model has a single order parameter, the density of particles. Our main result is a description at the level of the central limit theorem (CLT) of the fluctuations of the density of particles with respect to the NESS. We show that, in dimension $d \le 3$ and under a near-equilibrium condition, the scaling limit of these fluctuations is described by a Gaussian process, which is a mixture of a white noise and a massive Gaussian free field, and in particular it presents non-local spatial correlations. These non-local correlations are a signature of NESS, and their precise description is one of the main goals of a mathematical treatment of NESS. For the model considered here, these non-local correlations were predicted in [1].

At the level of the law of large numbers and the large deviations principle, the so-called *macroscopic fluctuation theory* (MFT) provides a fairly complete description of the fluctuations of the density of particles with respect to the NESS, see [3, 22]. However, at the level of the CLT, a description of the fluctuations is only available for a handful of models, [13, 14, 21, 25]. Although we prove our main result only for an example of a reaction-diffusion model, we claim that our framework can be used to derive a CLT for NESS of general driven diffusive models in dimensions $d \le 3$ under a near-equilibrium condition. The restriction on the dimension is necessary, but technical, while the near-equilibrium condition is necessary in the following sense. For reaction-diffusion models with cubic interactions, as the one considered in [9], a phase transition appears at bifurcation points of the effective reaction function defined in (2.3). Since our methodology also applies for the model considered in [9], it is natural to expect a restriction in terms of the parameters of the model.

The proof of the CLT for NESS is based on an explicit estimate of the *relative entropy* between the NESS and a product Bernoulli measure, valid for any dimension *d*. Our proof uses Yau’s relative entropy method [26], recently improved in [18] and that yields quantitative estimates for the convergence results. In dimension $d=1$, the entropy estimate is uniform on the size of the system. In dimensions $d \ge 2$, the estimate is not uniform on the size of the system. As a consequence of our results we are also able to show that the fluctuations of the NESS are absolutely continuous with respect to a white noise for any $d\le 3$.

Since our entropy estimates are explicit, they allow *quantitative* estimates on the law of large numbers for the NESS in every dimension $d \ge 1$. As far as we know, these estimates are the first example in the literature of what we call *quantitative hydrostatics*. Using the translation invariance of the NESS, we also show that in boxes of mesoscopic size, the structure of the NESS is indistinguishable from a Bernoulli product measure, a fact known in the literature as *local equilibrium*.

We point out that finite volume is essential for our methodology. In order to consider the process evolving on the infinite lattice ${\mathbb {Z}}^d$, new ideas are required. Reaction-diffusion processes in ${\mathbb {Z}}^d$ very similar to the ones considered here have been studied in [7, 8, 11], but in these works, the diffusion part is scaled in such a way that the system can be understood as a perturbation of the classical contact process, which opens the way to powerful renormalization arguments.

### Outline

In Sect. 2 we present the reaction-diffusion model we consider here and we state our main results. The main objective of Sect. 3 is to present a complete proof of Theorem 2.4, which is the estimate of the relative entropy of the NESS with respect to the Bernoulli product measure ${\nu _{\!\rho _*}^n}$, where ${\rho _*}$ is the unique zero in [0, 1] of the function *F* defined in (2.3). The proof combines Yau’s inequality, reviewed in Sect. 3.1, with the log-Sobolev inequality, reviewed in Sect. 3.2, and with Lemma 3.3, introduced in [18] and called *main lemma*. We present here a complete proof of the main lemma for two reasons. First, in our setting we only use translation-invariant reference measures, which makes the proof of Theorem 3.3 easier to follow in our context. And second, we need to keep track of all constants in the estimates, so that later on we can tune the parameter $\lambda$ to make the constants in Lemma 3.3 small enough. With the entropy estimate, the proof of Theorems 2.3 and 2.6 can be completed. In Sect. 4 we prove Theorem 2.7. In order to prove it, we use the *dynamical approach* introduced in [3] and used in [12] to derive a large deviations principle for the NESS of boundary-driven diffusive systems. In dimension $d=1$, the idea is simple. The entropy estimate of Theorem 2.4 implies tightness of the fluctuations of the density of particles around its hydrostatic limit. Moreover, any convergent subsequence satisfies the hypothesis of Theorem 2.4 in [18]. From these two facts, the proof of Theorem 2.7 follows as in [14]. In dimensions $d=2, 3$, the entropy estimate of Theorem 2.4 is not good enough to imply tightness directly. Therefore, we use a more indirect approach, based on Duhamel’s formula for solutions of the SPDE (4.2).

## Model and statement of results

Let $n \in {\mathbb {N}}$ be a scaling parameter and let ${{\mathbb {T}}^{d}_{n}}:= {\mathbb {Z}}^d / n {\mathbb {Z}}^d$ be the discrete torus of size *n* and dimension *d*. Let ${\Omega _n}:= \{0,1\}^{{\mathbb {T}}^{d}_{n}}$ be the state space of a Markov chain $({\eta ^n(t)}; t \ge 0)$ to be described below. The elements $x \in {{\mathbb {T}}^{d}_{n}}$ are called *sites* and the elements $\eta = (\eta _x; x \in {{\mathbb {T}}^{d}_{n}}) \in {\Omega _n}$ are called *particle configurations*. We say that the configuration $\eta \in {\Omega _n}$ has a particle at site $x \in {{\mathbb {T}}^{d}_{n}}$ if $\eta _x =1$. If $\eta _x =0$, we say that the site *x* is *empty*.

For $x,y \in {{\mathbb {T}}^{d}_{n}}$ and $\eta \in {\Omega _n}$, let $\eta ^{x,y} \in {\Omega _n}$ be given by

$$\begin{aligned} \eta ^{x,y}_z:=\left\{ \begin{array}{rcl} \eta _y &{}; &{} z =x,\\ \eta _x &{}; &{} z =y,\\ \eta _z &{}; &{} z \ne x,y. \end{array}\right. \end{aligned}$$

In other words, $\eta ^{x,y}$ is the particle configuration obtained from $\eta$ by exchanging the values of $\eta _x$ and $\eta _y$. For $f: {\Omega _n}\rightarrow {\mathbb {R}}$ and $x,y \in {{\mathbb {T}}^{d}_{n}}$, let $\nabla _{\!x,y} f: {\Omega _n}\rightarrow {\mathbb {R}}$ be given by

$$\begin{aligned} \nabla _{\!x,y} f(\eta ):= f(\eta ^{x,y}) -f(\eta ) \end{aligned}$$

for every $$\eta \in {\Omega _n}$$.

For $x \in {{\mathbb {T}}^{d}_{n}}$ and $\eta \in {\Omega _n}$, let $\eta ^x \in {\Omega _n}$ be given by

$$\begin{aligned} \eta ^{x}_z:=\left\{ \begin{array}{ccl} 1-\eta _x &{}; &{} z =x,\\ \eta _z &{}; &{} z \ne x, \end{array}\right. \end{aligned}$$

that is, $\eta ^x$ is the particle configuration obtained from $\eta$ by changing the value of $\eta _x$. For $f: {\Omega _n}\rightarrow {\mathbb {R}}$ and $x \in {{\mathbb {T}}^{d}_{n}}$, let $\nabla _x f: {\Omega _n}\rightarrow {\mathbb {R}}$ be given by

$$\begin{aligned} \nabla _x f(\eta ):= f(\eta ^x) -f (\eta ) \end{aligned}$$

for every $$\eta \in {\Omega _n}$$.

Let $x,y \in {{\mathbb {T}}^{d}_{n}}$. We say that $x \sim y$ if $|x_1-y_1|+\dots +|x_d-y_d|=1$. Let $a,b > 0$ and $\lambda >-a$. For each $x \in {{\mathbb {T}}^{d}_{n}}$, let $c_x = c_x(a,b,\lambda ,d): {\Omega _n}\rightarrow [0,\infty )$ be given by

2.1 $$\begin{aligned} c_x(\eta ) := \Big (a+ \frac{\lambda }{2d} \sum _{\begin{array}{c} y \in {{\mathbb {T}}^{d}_{n}}\\ y \sim x \end{array}} \eta _y\Big ) (1-\eta _x) +b \eta _x \end{aligned}$$

for every $\eta \in {\Omega _n}$. Observe that the conditions on *a*, *b* and $\lambda$ imply that $c_x(\eta ) \ge {\varepsilon _0}>0$ for every $x \in {{\mathbb {T}}^{d}_{n}}$ and every $\eta \in {\Omega _n}$, where ${\varepsilon _0}:= \min \{a, a+\lambda ,b\}$. This condition makes the Markov chain defined below *irreducible*. Later on the parameter $\lambda$ will be chosen smaller than some constant $\lambda _c= \lambda _c(a,b,d)$. For each $f: {\Omega _n}\rightarrow {\mathbb {R}}$, let $L_n f: {\Omega _n}\rightarrow {\mathbb {R}}$ be given by^1^

2.2 $$\begin{aligned} L_n f := n^2 {\!\!\sum _{\begin{array}{c} x, y \in {{\mathbb {T}}^{d}_{n}}\\ x \sim y \end{array}}\!\!}\nabla _{\!x,y} f + \sum _{x \in {{\mathbb {T}}^{d}_{n}}} c_x \nabla _x f. \end{aligned}$$

The linear operator $L_n$ defined in this way turns out to be the generator of a Markov chain in ${\Omega _n}$ that we denote by $({\eta ^n(t)}; t \ge 0)$. The sequence of chains $({\eta ^n(t)}; t \ge 0)_{n \in {\mathbb {N}}}$ is an example of what is known in the literature as a *reaction-diffusion model*.

As pointed out above, under the condition ${\varepsilon _0}>0$, the chain $({\eta ^n(t)}; t \ge 0)$ is irreducible, and therefore it has a unique invariant measure that will be denoted by ${\mu _{\textrm{ss}}^n}$. For $\lambda \ne 0$, the measure ${\mu _{\textrm{ss}}^n}$ is not reversible with respect to $({\eta ^n(t)}; t \ge 0)$. For this reason, we say that the measure ${\mu _{\textrm{ss}}^n}$ is a *non-equilibrium stationary state* (NESS). Our main goal is the description of the scaling limits of the density of particles with respect to the measures $({\mu _{\textrm{ss}}^n}; n \in {\mathbb {N}})$. For the moment we will not be very specific about what we understand by the density of particles; a proper definition will be included where needed.

### Notation

We will denote by ${\mathbb {P}}^n$ the law of $({\eta ^n(t)}; t \ge 0)$ on the space ${\mathcal {D}}([0,\infty ), {\Omega _n})$ of *càdlàg* trajectories, and we will denote by ${\mathbb {E}}^n$ the expectation with respect to ${\mathbb {P}}^n$. Whenever we need to specify the initial law $\mu ^n$ of the chain $({\eta ^n(t)}; t \ge 0)$, we will use the notations ${\mathbb {P}}^n_{\!\mu ^n}$, ${\mathbb {E}}^n_{\mu ^n}$. We will denote by *C* a finite and positive constant depending only on *a*, *b*, *d*, which may change from line to line.

### Hydrodynamic limit and hydrostatic limit

The generator $L_n$ combines an *exclusion dynamics*, corresponding to the operator $L_n^{\textrm{ex}}$ given by

$$\begin{aligned} L_n^{\textrm{ex}} f:= n^2 {\!\!\sum _{\begin{array}{c} x, y \in {{\mathbb {T}}^{d}_{n}}\\ x \sim y \end{array}}\!\!}\nabla _{\!x,y} f, \end{aligned}$$

with a *reaction dynamics*, corresponding to the operator $L_n^{\textrm{r}}$ given by

$$\begin{aligned} L_n^{\textrm{r}} f:= \sum _{x \in {{\mathbb {T}}^{d}_{n}}} c_x \nabla _x f. \end{aligned}$$

Observe that the reaction rates defined in (2.1) correspond to a *contact process* with an additional creation term avoiding absorption at density zero. The reaction-diffusion model was introduced in [9] with a reaction term corresponding to a stochastic Ising model. The key observation of [9] is the following. In order that both parts of the dynamics, the exclusion part and the reaction part, to have a non-trivial effect on the scaling limits of the density of particles, one must include a factor $n^2$ in front of the exclusion part of the dynamics, as in the definition of $L_n$ given in (2.2). In [9] the authors derive the so-called *hydrodynamic limit* of the reaction-diffusion model, which we now describe. For $\rho \in [0,1]$, let ${\nu _{\rho }^{n}}$ be the Bernoulli product measure in ${\Omega _n}$ of density $\rho$:

$$\begin{aligned} {\nu _{\rho }^{n}}(\eta ):= \prod _{x \in {{\mathbb {T}}^{d}_{n}}} \big ( \eta _x \rho +(1-\eta _x)(1-\rho )\big ) \end{aligned}$$

for every $\eta \in {\Omega _n}$. Fix $x \in {{\mathbb {T}}^{d}_{n}}$ and let $F: [0,1] \rightarrow {\mathbb {R}}$ be given by

2.3 $$\begin{aligned} F(\rho ) := \int L_n \eta _x\, d {\nu _{\rho }^{n}}\end{aligned}$$

for every $\rho \in [0,1]$. Since the operator $L_n$ and the measures $({\nu _{\rho }^{n}}; \rho \in [0,1])$ are translation invariant, *F* does not depend on *x*. Observe that

$$\begin{aligned} L_n \eta _x= & {} n^2 {\sum _{\begin{array}{c} y \in {{\mathbb {T}}^{d}_{n}}\\ y \sim x \end{array}}}(\eta _y -\eta _x) + c_x(\eta ) (1-2\eta _x) = n^2 {\sum _{\begin{array}{c} y \in {{\mathbb {T}}^{d}_{n}}\\ y \sim x \end{array}}}(\eta _y -\eta _x) \\{} & {} + \Big (a+ \frac{\lambda }{2d} {\sum _{\begin{array}{c} y \in {{\mathbb {T}}^{d}_{n}}\\ y \sim x \end{array}}}\eta _y \Big ) (1-\eta _x) - b\eta _x. \end{aligned}$$

Therefore,

2.4 $$\begin{aligned} F(\rho ) = (a+\lambda \rho ) (1-\rho ) - b \rho \end{aligned}$$

for every $\rho \in [0,1]$. We will also need to introduce the function $G: [0,1] \rightarrow {\mathbb {R}}$ given by

2.5 $$\begin{aligned} G(\rho ) := (a+\lambda \rho ) (1-\rho ) + b \rho \end{aligned}$$

for every $\rho \in [0,1]$. Observe that $G(\rho ) = F(\rho ) + 2b \rho$, although this relation is not relevant in what follows. We denote by ${\mathbb {T}}^d$ the d-dimensional torus. Let ${\mathcal {C}}({\mathbb {T}}^d; {\mathbb {R}})$ be the set of continuous functions $f: {\mathbb {T}}^d \rightarrow {\mathbb {R}}$ and let $(\pi _t^n; t \ge 0)$ be the family of measures defined by duality as

$$\begin{aligned} \pi _t^n(f):= \frac{1}{n^d} {\sum _{x \in {{\mathbb {T}}^{d}_{n}}}}{\eta _x^n(t)}f\big (\tfrac{x}{n}\big ) \end{aligned}$$

for every $f \in {\mathcal {C}}({\mathbb {T}}^d; {\mathbb {R}})$. We have that

#### Proposition 2.1

(Hydrodynamic limit [9, 20]) Let $(\mu ^n; n \in {\mathbb {N}})$ be a sequence of probability measures in ${\Omega _n}$ and let $u_0: {\mathbb {T}}^d \rightarrow [0,1]$ be a given measurable function. Assume that for every $f \in {\mathcal {C}}({\mathbb {T}}^d; {\mathbb {R}})$,

$$\begin{aligned} \lim _{n \rightarrow \infty } \frac{1}{n^d} {\sum _{x \in {{\mathbb {T}}^{d}_{n}}}}\eta _x f \big ( \tfrac{x}{n}\big ) = \int _{{\mathbb {T}}^d} u_0(x) f(x) dx \end{aligned}$$

in probability with respect to $(\mu ^n; n \in {\mathbb {N}})$. For every $f \in {\mathcal {C}}({\mathbb {T}}^d; {\mathbb {R}})$ and every $t \ge 0$,

$$\begin{aligned} \lim _{n \rightarrow \infty } \pi _t^n(f) = \int _{{\mathbb {T}}^d} u_t(x) f(x) dx \end{aligned}$$

in probability with respect to ${\mathbb {P}}^n_{\mu ^n}$, where $(u_t; t \ge 0)$ is the solution of the hydrodynamic equation

2.6 $$\begin{aligned} \partial _t u = \Delta u + F(u) \end{aligned}$$

with initial condition $u_0$.

This result is known in the literature as the hydrodynamic limit of the reaction-diffusion model $({\eta ^n(t)}; t \ge 0)_{n \in {\mathbb {N}}}$. The hydrodynamic equation (2.6) is an example of a *reaction-diffusion equation*. The intuition behind the definition of the function *F* appearing in (2.6) is the following. If we look at the dynamics on a box of size $1 \ll \ell \ll n$, the exclusion dynamics is much faster than the reaction dynamics; in fact, the exclusion dynamics takes times of order ${\mathcal {O}}(\frac{\ell ^2}{n^2})$ in order to equilibrate the density of particles on a box of size $\ell$, and the first jump of the reaction dynamics happens after times of order $\ell ^{-d}$. Therefore, if $\ell \ll n^{\frac{2}{2+d}}$, the exclusion dynamics equilibrates the density of particles between consecutive jumps of the reaction dynamics. Since the Bernoulli product measures are invariant under the exclusion dynamics, it is reasonable to assume that at least on boxes of size $\ell \ll n^{\frac{2}{2+d}}$, the law of the process is close to product. Therefore it makes sense to define the function *F* as the average reaction rate with respect to ${\nu _{\rho }^{n}}$. This intuition will be made rigorous for the invariant measure ${\mu _{\textrm{ss}}^n}$ in Theorem 2.6 below. Theorem 2.6 also shows that the size $\ell$ of the boxes on which this product approximation is accurate much larger than $n^{\frac{2}{2+d}}$.

Observe that $F(0) =a>0$, $F(1) =-b<0$ and *F* is a polynomial of degree 2. Therefore, *F* has a unique zero ${\rho _*}$ in [0, 1], which satisfies $0< {\rho _*}< 1$ and $F'({\rho _*}) <0$, from where we conclude that ${\rho _*}$ is also a stable stationary solution to (2.6). The density ${\rho _*}$ can be explicitly computed, but its exact formula plays no role in our proofs; we will only use the property

$$\begin{aligned} \lim _{\lambda \rightarrow 0} {\rho _*}= \frac{a}{a+b} \in (0,1). \end{aligned}$$

This limit can be directly verified from the explicit formula for ${\rho _*}$ or it can be deduced from the fact that $F(\rho ) \rightarrow a -\rho (a+b)$ as $\lambda \rightarrow 0$.

In view of the stationarity properties of the hydrodynamic equation (2.6), it is reasonable to postulate that, with respect to ${\mu _{\textrm{ss}}^n}$, the density of particles is close to ${\rho _*}$. However, a proof of this claim requires a non-trivial exchange of limits. Such exchange of limits has been justified in [22], elaborating over an idea introduced in [12, 23]:

#### Proposition 2.2

(Hydrostatic limit) For every $f \in {\mathcal {C}}({\mathbb {T}}^d; {\mathbb {R}})$,

$$\begin{aligned} \lim _{n \rightarrow \infty } \frac{1}{n^d} {\sum _{x \in {{\mathbb {T}}^{d}_{n}}}}\eta _x f \big ( \tfrac{x}{n} \big ) = \int _{{\mathbb {T}}^d} {\rho _*}f(x) dx \end{aligned}$$

in probability with respect to $\{{\mu _{\textrm{ss}}^n}; n \in {\mathbb {N}}\}$.

Our first result is a quantitative version of Proposition 2.2. For $n,d \in {\mathbb {N}}$, let $g_d(n)$ be defined as

2.7 $$\begin{aligned} g_d(n):=\left\{ \begin{array}{ccr} n &{} ; &{} d=1,\\ \log n &{} ; &{} d=2, \\ 1 &{} ; &{} d \ge 3. \end{array}\right. \end{aligned}$$

Observe that $g_d(n)$ corresponds to the order of magnitude of the Green’s function at 0 of a simple symmetric random walk absorbed at the boundary of a box of size *n*, centered at the origin.

#### Theorem 2.3

There exist $\lambda _c = \lambda _c(a,b,d)$ positive and $C=C(a,b,d)$ finite such that for every $f \in {\mathcal {C}}({\mathbb {T}}^d; {\mathbb {R}})$, every $n \in {\mathbb {N}}$ and every $\lambda \in [-\lambda _c,\lambda _c]$,

$$\begin{aligned} \int \Big (\frac{1}{n^d} {\sum _{x \in {{\mathbb {T}}^{d}_{n}}}}(\eta _x - {\rho _*}) f \big ( \tfrac{x}{n} \big ) \Big )^2 {\mu _{\textrm{ss}}^n}(d \eta ) \le \frac{C g_d(n)}{n^2} \cdot \frac{1}{n^d} {\sum _{x \in {{\mathbb {T}}^{d}_{n}}}}f \big (\tfrac{x}{n}\big )^2. \end{aligned}$$

For every probability measure $\mu$ in $\Omega _n$ and every density *f* with respect to $\mu$, let $H(f; \mu )$ denote the relative entropy of *f* with respect to $\mu$:

$$\begin{aligned} H(f; \mu ):= \int f \log f d \mu . \end{aligned}$$

Theorem 2.3 is a consequence of the following estimate:

#### Theorem 2.4

Let ${f_{\textrm{ss}}^n}$ be the density of ${\mu _{\textrm{ss}}^n}$ with respect to ${\nu _{\!\rho _*}^n}$. There exist $\lambda _c = \lambda _c(a,b,d)$ positive and $C = C(a,b,d)$ finite such that

$$\begin{aligned} H( {f_{\textrm{ss}}^n}; {\nu _{\!\rho _*}^n}) \le C n^{d-2} g_d(n) \end{aligned}$$

for every $n \in {\mathbb {N}}$ and every $\lambda \in [-\lambda _c,\lambda _c]$.

#### Remark 2.5

The value of $\lambda _c$ is the same in Theorems 2.3 and 2.4, but the value of *C* can be different.

Given two probability measures $\mu$, $\nu$ in $\Omega _n$, the relative entropy of $\mu$ with respect to $\nu$ is defined as $H( \frac{d \mu }{d \nu }; \nu )$ if $\mu$ is absolutely continuous with respect to $\nu$ and $+\infty$ otherwise. Although relative entropy is not a distance (it is not even symmetric), it is widely used in the literature as a measure of closeness between probability measures. Another popular way to measure the closeness of two probability measures is through the *total variation distance*. The total variation distance between $\mu$ and $\nu$ is defined as

$$\begin{aligned} d_{{{\,\textrm{TV}\,}}}(\mu ,\nu ):= \frac{1}{2} \sum _{\eta \in \Omega _n} |\mu (\eta )-\nu (\eta )|. \end{aligned}$$

Total variation and relative entropy are related by *Pinsker’s inequality*:

$$\begin{aligned} 2 d_{{{\,\textrm{TV}\,}}}(\mu ,\nu )^2 \le H \Big (\frac{d\mu }{d \nu }; \nu \Big ). \end{aligned}$$

Observe that the relative entropy bound of Theorem 2.4 is not strong enough to conclude that the measures ${\mu _{\textrm{ss}}^n}$ and ${\nu _{\!\rho _*}^n}$ are close. This is actually expected, since we will see in Theorem 3.4 below that the scaling limits of the density of particles are different under ${\mu _{\textrm{ss}}^n}$ and ${\nu _{\!\rho _*}^n}$. Nevertheless, we will prove that the bound of Theorem 2.4 is good enough to show a strong version of what is known in the literature as *conservation of local equilibrium*, namely, that restricted to boxes of mesoscopic size, the measures ${\mu _{\textrm{ss}}^n}$ and ${\nu _{\!\rho _*}^n}$ are close in total variation. In order to be precise, we need a few definitions. Let ${\mathcal {P}}: {\mathbb {Z}}^d \rightarrow {{\mathbb {T}}^{d}_{n}}$ be the universal cover of ${{\mathbb {T}}^{d}_{n}}$. For $R \in {\mathbb {N}}$, let $B_R:= \{x \in {\mathbb {Z}}^ d; |x_i| \le R, i=1,\dots ,d\}$. For $n > 2R+1$, let $\Pi _R: \Omega _n \rightarrow \{0,1\}^{B_R}$ be the canonical projection: $(\Pi _R \eta )_x = \eta _{{\mathcal {P}}(x)}$ for every $x \in B_R$. Let ${\mu _{\textrm{ss}}^{n,R}}$ be the push-forward of ${\mu _{\textrm{ss}}^n}$ under $\Pi _R$ and let ${\nu _{\rho _*}^{n, R}}$ be the push-forward of ${\nu _{\!\rho _*}^n}$ under $\Pi _R$. We will prove the following result:

#### Theorem 2.6

If

$$\begin{aligned} \frac{R_n g_d(n)^{1/d}}{n^{2/d}} \rightarrow 0 \text { as } n \rightarrow \infty \end{aligned}$$

then

$$\begin{aligned} d_{{{\,\textrm{TV}\,}}} ( {\mu _{\textrm{ss}}^{n,R_n}}, {\nu _{\rho _*}^{n, R_n}} ) \rightarrow 0 \text { as } n \rightarrow \infty . \end{aligned}$$

This theorem states that on a window of mesoscopic size $R_n$, the NESS ${\mu _{\textrm{ss}}^n}$ is asymptotically indistinguishable from a Bernoulli product measure of density ${\rho _*}$.

### Fluctuations and CLT for NESS

Observe that Theorem 2.3 can be understood as a law of large numbers for the density of particles with respect to $({\mu _{\textrm{ss}}^n}; n \in {\mathbb {N}})$. Therefore, it is natural to study the fluctuations of the density of particles around its hydrodynamic limit. The *density fluctuation field* is the function $X^n: {\Omega _n}\rightarrow {\mathcal {S}}'({\mathbb {T}}^d)$ defined by duality as

$$\begin{aligned} X^n(\eta , f): = \frac{1}{n^{d/2}} {\sum _{x \in {{\mathbb {T}}^{d}_{n}}}}(\eta _x - {\rho _*}) f\big ( \tfrac{x}{n} \big ) \end{aligned}$$

for every $f \in {\mathcal {C}}^\infty ({\mathbb {T}}^d; {\mathbb {R}})$ and every $\eta \in {\Omega _n}$. Although $X^n$ is well defined as a signed measure in ${\mathbb {T}}^d$, it is more convenient to think about $X^n$ as a *random distribution*. In a probabilistic context, on which ${\mathcal {L}}^2$-norms are related to variances, *Sobolev spaces* are a specially convenient choice of topology for the image of $X^n$. It will be convenient to define Sobolev spaces in terms of Fourier transforms. For $f \in {\mathcal {L}}^1({\mathbb {T}}^d)$, let $\hat{f}: {\mathbb {Z}}^d \rightarrow {\mathbb {C}}$ be given by

2.8 $$\begin{aligned} \hat{f} (k) := \int _{{\mathbb {T}}^d} e^{-2 \pi i k x} f(x) dx \end{aligned}$$

for every $k \in {\mathbb {Z}}^d$, that is, $\hat{f}$ is the *Fourier transform* of *f*. For $k=(k_1,\dots k_d) \in {\mathbb {Z}}^d$, let us write $\Vert k\Vert := (k_1^2+\dots +k_d^2)^{1/2}$. For $f \in {\mathcal {C}}^\infty ({\mathbb {T}}^d; {\mathbb {R}})$ and $m \in {\mathbb {N}}$, let

$$\begin{aligned} \Vert f\Vert _{{\mathcal {H}}^m}:= \Big ( \sum _{k \in {\mathbb {Z}}^d} | \hat{f} (k)|^2 (1+ \Vert k\Vert ^2)^m\Big )^{1/2}. \end{aligned}$$

Observe that $\Vert f\Vert _{{\mathcal {H}}^m}<+\infty$ for every $m \in {\mathbb {N}}$ and every $f \in {\mathcal {C}}^\infty ({\mathbb {T}}^d; {\mathbb {R}})$, and observe that the space $(C^\infty ({\mathbb {T}}^d; {\mathbb {R}}),\Vert \cdot \Vert _{{\mathcal {H}}^m})$ is pre-Hilbert. The so-called *Sobolev space* ${\mathcal {H}}^m={\mathcal {H}}^m({\mathbb {T}}^d)$ of order *m* is defined as the closure of ${\mathcal {C}}^\infty ({\mathbb {T}}^d; {\mathbb {R}})$ with respect to $\Vert \cdot \Vert _{{\mathcal {H}}^m}$. All the spaces ${\mathcal {H}}^m$ are Hilbert spaces, and by Parseval’s identity, ${\mathcal {H}}^0 = {\mathcal {L}}^2({\mathbb {T}}^d)$. Moreover, for every $m \ne 0$, the spaces ${\mathcal {H}}^m, {\mathcal {L}}^2({\mathbb {T}}^d)$ and ${\mathcal {H}}^{-m}$ form a *Gelfand triple*. Observe as well that ${\mathcal {H}}^{m'}$ is compactly embedded in ${\mathcal {H}}^m$ if $m < m'$. By the Sobolev embedding theorem, the Dirac $\delta$ distribution belongs to ${\mathcal {H}}^{-m}$ for every $m > d/2$, and we see that $X^n$ is a random variable in ${\mathcal {H}}^{-m}$ for every $m > d/2$.

Let $\chi ({\rho _*}):= {\rho _*}(1-{\rho _*})$ be the *mobility* of the reaction-diffusion model. This quantity is one of the thermodynamic variables appearing in MFT [5]. Now we can state our main result:

#### Theorem 2.7

Let $d \le 3$ and let $\eta$ have law ${\mu _{\textrm{ss}}^n}$. There exists $\lambda _c=\lambda _c(a,b,d) >0$ such that for every $\lambda \in [-\lambda _c,\lambda _c]$,

$$\begin{aligned} \lim _{n \rightarrow \infty } X^n = X_\infty \end{aligned}$$

in law with respect to the topology of ${\mathcal {H}}^{-m}$ for

$$\begin{aligned} m > \left\{ \begin{array}{ccl} 1/2 &{}; &{} d=1,\\ 3 &{}; &{} d=2,\\ 9/2 &{}; &{} d =3, \end{array}\right. \end{aligned}$$

where $X_\infty$ is a centered Gaussian process of variance given by

2.9 $$\begin{aligned} {\mathbb {E}}[X_\infty (f)^2] = \sum _{k \in {\mathbb {Z}}^d} \big |\hat{f}(k)\big |^2 \Big (\chi ({\rho _*}) + \frac{G({\rho _*}) + 2 F'({\rho _*}) \chi ({\rho _*})}{8 \pi ^2 \Vert k\Vert ^2 - 2 F' ({\rho _*})}\Big ). \end{aligned}$$

for every $$f \in {\mathcal {C}}^\infty ({\mathbb {T}}^d; {\mathbb {R}})$$.

#### Remark 2.8

Observe that if $\lambda =0$, then $X_\infty$ is a white noise of variance $\chi ({\rho _*})$. The general expression in (2.9) can be guessed from fluctuating hydrodynamics, see the discussion after (SHE).

## The relative entropy method

In this section we will prove Theorem 2.4 and we will use it to prove Theorem 2.3. We will use Yau’s *relative entropy method*, introduced in [26]; see Chapter 6 of [19] for a review. We will use the approach of [18].

### Yau’s inequality

The *carré du champ* associated to the operator $L_n$ is the bilinear operator $\Gamma _n$ given by

$$\begin{aligned} \Gamma _n (f,g):= L_n(fg) - f L_n g - g L_n f \end{aligned}$$

for every $f,g: {\Omega _n}\rightarrow {\mathbb {R}}$. As usual in functional analysis, we will use the notation $\Gamma _n (f):= \Gamma _n(f,f)$. We will also define the *carrés du champ* associated to the operators $L_n^{\textrm{ex}}$, $L_n^{\textrm{r}}$:

$$\begin{aligned}{} & {} \Gamma _n^{\textrm{ex}} (f,g):= L_n^{\textrm{ex}}(fg) - fL_n^{\textrm{ex}} g - g L_n^{\textrm{ex}}f, \\{} & {} \Gamma _n^{\textrm{r}}(f,g):= L_n^{\textrm{r}} (fg) -f L_n^{\textrm{r}} g - g L_n^{\textrm{r}} f \end{aligned}$$

for every $$f,g: {\Omega _n}\rightarrow {\mathbb {R}}$$.

Let $L_n^*$ be the adjoint of $L_n$ with respect to ${\mathcal {L}}^2({\nu _{\!\rho _*}^n})$. Observe that ${\nu _{\!\rho _*}^n}$ is reversible under $L_n^{\textrm{ex}}$, that is, $(L_n^{\textrm{ex}})^*= L_n^{\textrm{ex}}$. Therefore, $L_n^*= L_n^{\textrm{ex}} + (L_n^{\textrm{r}})^*$. The operator $(L_n^{\textrm{r}})^*$ can be computed explicitly in terms of ${\nu _{\!\rho _*}^n}$:

3.1 $$\begin{aligned} (L_n^{\textrm{r}})^*f(\eta ) = {\sum _{x \in {{\mathbb {T}}^{d}_{n}}}}\Big ( c_x(\eta ^x) \frac{{\nu _{\!\rho _*}^n}(\eta ^x)}{{\nu _{\!\rho _*}^n}(\eta )}f(\eta ^x) - c_x(\eta ) f(\eta )\Big ). \end{aligned}$$

A more explicit form can be obtained by observing that

$$\begin{aligned} \frac{{\nu _{\!\rho _*}^n}(\eta ^x)}{{\nu _{\!\rho _*}^n}(\eta )} = \frac{\eta _x(1-{\rho _*})}{{\rho _*}} +\frac{(1-\eta _x){\rho _*}}{1-{\rho _*}}. \end{aligned}$$

We will use this identity to compute $L_n^*\textbf{1}$, where $\textbf{1}$ is the constant function equal to 1.

Since we already know that the density of particles under ${\mu _{\textrm{ss}}^n}$ is approximately equal to ${\rho _*}$, it is reasonable to start the chain $({\eta ^n(t)}; t \ge 0)$ from the initial measure ${\nu _{\!\rho _*}^n}$. Let $f_t^n$ be the density with respect to ${\nu _{\!\rho _*}^n}$ of the law of ${\eta ^n(t)}$ under ${\mathbb {P}}_{{\nu _{\!\rho _*}^n}}^n$. Let

$$\begin{aligned} H_n(t):= H (f_t^n; {\nu _{\!\rho _*}^n}) \end{aligned}$$

be the *relative entropy* of the law of ${\eta ^n(t)}$ under ${\mathbb {P}}_{{\nu _{\!\rho _*}^n}}^n$ with respect to ${\nu _{\!\rho _*}^n}$. The so-called *Yau’s inequality* [18, 26], states that

3.2 $$\begin{aligned} H_n'(t) \le - \int {\Gamma _n (\sqrt{f_t^n})} d {\nu _{\!\rho _*}^n}+ \int L_n^*\textbf{1} f_t^n d {\nu _{\!\rho _*}^n}. \end{aligned}$$

Since the chain $({\eta ^n(t)}; t \ge 0)$ is irreducible and the state space $\Omega _n$ is finite, $f_t^n \rightarrow {f_{\textrm{ss}}^n}$ as $t \rightarrow \infty$, and in particular, since the function $x\rightarrow x\log (x)$ is continuous, we have

$$\begin{aligned} H( {f_{\textrm{ss}}^n}; {\nu _{\!\rho _*}^n}) = \lim _{t \rightarrow \infty } H_n(t). \end{aligned}$$

Therefore, a uniform bound on $H_n(t)$ implies a bound on the relative entropy of ${\mu _{\textrm{ss}}^n}$ with respect to ${\nu _{\!\rho _*}^n}$. For every $\eta \in \Omega _n$ and every $x \in {{\mathbb {T}}^{d}_{n}}$, let us define

$$\begin{aligned} {\bar{\eta }}_x:= \eta _x - {\rho _*}. \end{aligned}$$

From (3.1),

3.3 $$\begin{aligned} L_n^*\textbf{1}&= {\sum _{x \in {{\mathbb {T}}^{d}_{n}}}}\Big \{\eta _x \Big ( \Big ( a+ \frac{\lambda }{2d} {\sum _{\begin{array}{c} y \in {{\mathbb {T}}^{d}_{n}}\\ y \sim x \end{array}}}\eta _y \Big ) \frac{1-{\rho _*}}{{\rho _*}} -b\Big ) +(1-\eta _x) \Big ( \frac{b {\rho _*}}{1-{\rho _*}} - \Big (a+\frac{\lambda }{2d} {\sum _{\begin{array}{c} y \in {{\mathbb {T}}^{d}_{n}}\\ y \sim x \end{array}}}\eta _y\Big )\Big ) \Big \}\nonumber \\&= {\sum _{x \in {{\mathbb {T}}^{d}_{n}}}}\Big ( \frac{\eta _x}{{\rho _*}} - \frac{1-\eta _x}{1-{\rho _*}} \Big ) \Big (\Big (a+\frac{\lambda }{2d}{\sum _{\begin{array}{c} y \in {{\mathbb {T}}^{d}_{n}}\\ y \sim x \end{array}}}\eta _y\Big ) (1-{\rho _*}) -b {\rho _*}\Big ) \nonumber \\&= {\sum _{x \in {{\mathbb {T}}^{d}_{n}}}}\frac{{\bar{\eta }}_x}{\chi ({\rho _*})}\Big ( F({\rho _*}) + \frac{\lambda }{2d} {\sum _{\begin{array}{c} y \in {{\mathbb {T}}^{d}_{n}}\\ y \sim x \end{array}}}{\bar{\eta }}_y (1-{\rho _*})\Big )\nonumber \\&= \frac{\lambda }{2d {\rho _*}} {\!\!\sum _{\begin{array}{c} x, y \in {{\mathbb {T}}^{d}_{n}}\\ x \sim y \end{array}}\!\!}{\bar{\eta }}_x {\bar{\eta }}_y, \end{aligned}$$

where in the last identity we used that $F({\rho _*}) =0$. Observe that $L_n^*\textbf{1}$ is a sum of monomials of degree two in the centered variables $({\bar{\eta }}_x; x \in {{\mathbb {T}}^{d}_{n}})$. For different choices of the reaction rates, monomials of degree three or higher can appear in this expression. The precise form of the hydrodynamic equation prevents the appearance of monomials of degree 1. The absence of monomials of degree 1 in the expression for $L_n^*\textbf{1}$ is fundamental in what follows; monomials of degree three of higher can also be handled with the methodology described below.

### The log-Sobolev inequality

In order to take full advantage of Yau’s inequality (3.2), it would be useful to have a lower bound for the integral $\int {\Gamma _n (\sqrt{f_t^n})} d {\nu _{\!\rho _*}^n}$ in terms of the relative entropy $H_n(t)$. This is exactly the content of the so-called *log-Sobolev inequality*, which we now explain. Let *L* be a Markov generator on $\Omega _n$ and let $\Gamma$ be the *carré du champ* associated to *L*. The *log-Sobolev constant* of *L* (or $\Gamma$) with respect to a measure $\mu$ in $\Omega _n$, is defined as

3.4 $$\begin{aligned} \alpha = \alpha ( \Gamma ; \mu ) := \inf \frac{\int {\Gamma (\sqrt{f})} d \mu }{ H ( f; \mu )}, \end{aligned}$$

where the infimum runs over all densities *f* with respect to $\mu$. It can be shown that $\alpha$ is always finite and that if *L* is irreducible, then $\alpha >0$. If *L* is irreducible and $\mu$ is equal to the invariant measure of *L*, then $\alpha$ can be used to estimate the speed of convergence to $\mu$ of the law of the chain generated by *L*, see [10] for a review.

For the generator $L_n$ defined in (2.2) and the measure ${\nu _{\!\rho _*}^n}$, the log-Sobolev constant is bounded from below by a positive constant that does not depend on *n*, a result known in the literature as the *log-Sobolev inequality*:

#### Lemma 3.1

(log-Sobolev inequality) For every $n \in {\mathbb {N}}$, every $\rho \in (0,1)$, every $a,b >0$ and every $\lambda >-a$,

$$\begin{aligned} \alpha ( \Gamma _n^{\textrm{r}}; {\nu _{\rho }^{n}}) \ge \frac{ \varepsilon _0 |1-2\rho |}{2\rho (1-\rho )\big |\log \frac{\rho }{1-\rho }\big |}=: \kappa (\rho )^{-1}, \end{aligned}$$

where $$\varepsilon _0:= \min \{a,a+\lambda , b\}$$.

#### Remark 3.2

By continuity, this lemma holds with right-hand side equal to $\varepsilon _0$ when $\rho = 1/2$.

#### Proof

This is a classical result in the literature, so we will only give a sketch of its proof. According to [10, Theorem A.2 and Lemma 3.2],

$$\begin{aligned} H( f; {\nu _{\rho }^{n}}) \le \frac{2 \rho (1-\rho ) \big | \log \frac{\rho }{1-\rho }\big |}{|1-2\rho |} {\sum _{x \in {{\mathbb {T}}^{d}_{n}}}}\int \big ( \nabla _x \sqrt{f} \big )^2 d {\nu _{\rho }^{n}}\end{aligned}$$

for every $\rho \in (0,1)$ and every density *f* with respect to ${\nu _{\rho }^{n}}$. Since $c_x(\eta ) \ge \varepsilon _0$ for every $x \in {{\mathbb {T}}^{d}_{n}}$ and every $\eta \in {\Omega _n}$,

$$\begin{aligned} \int \Gamma ^{\textrm{r}}_n {(\sqrt{f})} d {\nu _{\rho }^{n}}\ge \varepsilon _0 {\sum _{x \in {{\mathbb {T}}^{d}_{n}}}}\int \big ( \nabla _x \sqrt{f} \big )^2 d {\nu _{\rho }^{n}}\end{aligned}$$

for every density *f* with respect to ${\nu _{\rho }^{n}}$, which proves the lemma. $\square$

### The main lemma

In order to make an effective use of Yau’s inequality as stated in (3.2), we need to estimate the integral $\int L_n^*\textbf{1} f_t^n d {\nu _{\!\rho _*}^n}$ in terms of the relative entropy $H_n(t)$ and the *energy* $\int \Gamma _n {(\sqrt{f_t^n})} d {\nu _{\!\rho _*}^n}$. It turns out that the specific form of $f_t^n$ as the density of a Markov chain does not play any role in this estimation procedure. Therefore, we will estimate $\int L_n^*\textbf{1} f d {\nu _{\!\rho _*}^n}$ for arbitrary densities *f*. Moreover, it will be useful to consider $L_n^*\textbf{1}$ as a particular instance of the sum

3.5 $$\begin{aligned} V(g) := \sum _{i=1}^d {\sum _{x \in {{\mathbb {T}}^{d}_{n}}}}{\bar{\eta }}_x {\bar{\eta }}_{x+e_i} g_x^i, \end{aligned}$$

where $g=(g^1,\dots ,g^d): {{\mathbb {T}}^{d}_{n}}\rightarrow {\mathbb {R}}^d$ is a given function and where $\{e_1,\dots ,e_d\}$ denotes the canonical basis of ${{\mathbb {T}}^{d}_{n}}$. According to (3.3), $L_n^*\textbf{1}$ corresponds to the case $g_x^i= \frac{\lambda }{2d {\rho _*}}$. The so-called *main lemma*, introduced in Theorem 3.1 of [18], allows us to replace ${\bar{\eta }}_x {\bar{\eta }}_y$ by products of averages in boxes of mesoscopic size, with a cost controlled by the energy $\int \Gamma _n \sqrt{f_t^n} d {\nu _{\!\rho _*}^n}$. Since we need to be very precise about the dependence on $\lambda$ of the constants appearing in this lemma, we will present its proof. This proof follows very closely the proof in [18] and it can be omitted by the reader satisfied with a reference to [18]. In our setting, the proof of the main lemma is considerably simpler, so we take this opportunity to present the methodology of [18] in a clearer way. Recall the definition of $\kappa (\rho )$ given in Lemma 3.1 and let us define ${\mathcal {A}}(u):= u(1+u)$ for every $u \ge 0$.

#### Theorem 3.3

(Main lemma) There exists a constant $C= C(d)$ such that

$$\begin{aligned} \int V(g) f d {\nu _{\!\rho _*}^n}&\le \frac{1}{4} \int {\Gamma _n^{\textrm{ex}} (\sqrt{f})} d {\nu _{\!\rho _*}^n}+ C \kappa ({\rho _*}) {\mathcal {A}}(\Vert g\Vert _\infty ) \int { \Gamma _n^{\textrm{r}} (\sqrt{f})} d {\nu _{\!\rho _*}^n}\\&\quad + C {\mathcal {A}}(\Vert g\Vert _\infty ) n^{d-2} g_d(n) \end{aligned}$$

for every $n \in {\mathbb {N}}$, every $g: {{\mathbb {T}}^{d}_{n}}\rightarrow {\mathbb {R}}^d$ and every density *f* with respect to ${\nu _{\!\rho _*}^n}$.

#### Proof

For $\ell < n$ and $x \in {{\mathbb {T}}^{d}_{n}}$, let ${\mathbb {C}}_x^\ell$ the cube of vertex *x* and side $\ell$ given by

$$\begin{aligned} {\mathbb {C}}_x^\ell := \{y \in {{\mathbb {T}}^{d}_{n}}; y_i-x_i \in \{0,1,\dots ,\ell -1\} \text { for every } i \in \{1,\dots ,d\}\}. \end{aligned}$$

Let $p^\ell :{{\mathbb {T}}^{d}_{n}}\rightarrow [0,1]$ be the uniform measure in ${\mathbb {C}}_0^\ell$, that is, $p^\ell (x) = \ell ^{-d}\textbf{1}(x \in {\mathbb {C}}_0^\ell )$ for every $x \in {{\mathbb {T}}^{d}_{n}}$. For $\ell <n/2$, let $q^\ell : {{\mathbb {T}}^{d}_{n}}\rightarrow [0,1]$ be given by $q^\ell := p^\ell *p^\ell$, that is,

$$\begin{aligned} q^\ell (y):= {\sum _{x \in {{\mathbb {T}}^{d}_{n}}}}p^\ell (y-x) p^\ell (x) \end{aligned}$$

for every $y \in {{\mathbb {T}}^{d}_{n}}$. Observe that $q^\ell$ is supported on ${\mathbb {C}}^{2\ell -1}_0$.

For $\eta \in {\Omega _n}$, $x \in {{\mathbb {T}}^{d}_{n}}$ and $\ell <n/2$, let ${\bar{\eta }}_x^\ell \in {\mathbb {R}}$ be given by

$$\begin{aligned} {\bar{\eta }}_x^\ell := {\sum _{y \in {{\mathbb {T}}^{d}_{n}}}}q^\ell (y) {\bar{\eta }}_{x+y}. \end{aligned}$$

Let us define as well

$$\begin{aligned}{} & {} {\overset{\rightarrow }{\eta }}_{x}^{\ell }:= {\sum _{y \in {{\mathbb {T}}^{d}_{n}}}}p^\ell (y) {\bar{\eta }}_{x+y}, \quad {\overset{\leftarrow }{\eta }}_{x}^{\ell ,i}(g):= {\sum _{y \in {{\mathbb {T}}^{d}_{n}}}}p^\ell (y){\bar{\eta }}_{x-y} g_{x-y}^i, \\{} & {} V^\ell (g):= \sum _{i=1}^d {\sum _{x \in {{\mathbb {T}}^{d}_{n}}}}{\bar{\eta }}_x {\bar{\eta }}_{x+e_i}^\ell g_x^i. \end{aligned}$$

Thanks to our choice of the probability measure $q^\ell$, the sum $V^\ell (g)$ can be rewritten as

$$\begin{aligned} V^\ell (g) = \sum _{i=1}^d {\sum _{x \in {{\mathbb {T}}^{d}_{n}}}}{\overset{\leftarrow }{\eta }}_{x}^{\ell ,i}(g) {\overset{\rightarrow }{\eta }}_{x+e_i}^{\ell }. \end{aligned}$$

This identity will make the proof of Theorem 3.3 shorter. The idea is to estimate $\int (V(g) - V^\ell (g))f d {\nu _{\!\rho _*}^n}$ in terms of $\int {\Gamma _n^{\textrm{ex}} (\sqrt{f})} d {\nu _{\!\rho _*}^n}$. Observe that

$$\begin{aligned} V(g) - V^\ell (g) = \sum _{i=1}^d {\sum _{x \in {{\mathbb {T}}^{d}_{n}}}}{\bar{\eta }}_x ({\bar{\eta }}_{x+e_i} - {\bar{\eta }}_{x+e_i}^\ell ) g_x^i. \end{aligned}$$

Let us rewrite the difference ${\bar{\eta }}_x - {\bar{\eta }}_x^\ell$ as a linear combination of terms of the form ${\bar{\eta }}_z-{\bar{\eta }}_y$, where $y \sim z$. In order to do that, we will use *flows*.

Let ${\mathcal {E}}^d_n:= \{(x,y); x, y \in {{\mathbb {T}}^{d}_{n}}, x \sim y\}$ be the set of oriented edges of the periodic lattice ${{\mathbb {T}}^{d}_{n}}$. We say that a function $\phi : {\mathcal {E}}^d_n \rightarrow {\mathbb {R}}$ is a *flow* if $\phi (x,y) = - \phi (y,x)$ for every $(x,y) \in {\mathcal {E}}^d_n$. Let *p*, *q* be two probability measures in ${{\mathbb {T}}^{d}_{n}}$. We say that a flow $\phi$ *connects* *p* to *q* if

$$\begin{aligned} p(x)-q(x) = {\sum _{\begin{array}{c} y \in {{\mathbb {T}}^{d}_{n}}\\ y \sim x \end{array}}}\phi (x,y) \end{aligned}$$

for every $x \in {{\mathbb {T}}^{d}_{n}}$. In other words, $p-q = {{\,\textrm{div}\,}}\phi$. In that case, *p*, *q* and $\phi$ satisfy the *divergence formula*: for every $g: {{\mathbb {T}}^{d}_{n}}\rightarrow {\mathbb {R}}$,

3.6 $$\begin{aligned} {\sum _{x \in {{\mathbb {T}}^{d}_{n}}}}g(x)(p(x)-q(x)) = {\!\!\sum _{\begin{array}{c} x, y \in {{\mathbb {T}}^{d}_{n}}\\ x \sim y \end{array}}\!\!}\phi (x,y) (g(x)-g(y)). \end{aligned}$$

Recall the definition of $g_d(n)$ given in (2.7). Lemma 3.2 in [18] tells us that for every $\ell < n/2$ there exists a finite $C=C(d)$ and a flow $\phi ^\ell$ connecting the Dirac $\delta$ at $x=0$ to $q^\ell$ such that

i) $$\displaystyle {{\!\!\sum _{\begin{array}{c} x, y \in {{\mathbb {T}}^{d}_{n}}\\ x \sim y \end{array}}\!\!}\phi ^\ell (x,y)^2 \le C g_d(\ell )}$$,
ii) $\phi ^\ell (x,y)=0$ whenever $x \notin {\mathbb {C}}_0^{2\ell -1}$ or $y \notin {\mathbb {C}}_0^{2\ell -1}$.

Using the flow $\phi ^\ell$ given by Lemma 3.2 of [18], the translation invariance of the lattice ${{\mathbb {T}}^{d}_{n}}$ and the divergence formula (3.6), we see that

$$\begin{aligned} {\bar{\eta }}_x - {\bar{\eta }}_x^\ell = {{\!\!\sum _{\begin{array}{c} y, z \in {{\mathbb {T}}^{d}_{n}}\\ y \sim z \end{array}}\!\!}}\phi ^\ell (y,z) ({\bar{\eta }}_{x+y}-{\bar{\eta }}_{x+z}), \end{aligned}$$

from where

$$\begin{aligned}&\sum _{i=1}^d {\sum _{x \in {{\mathbb {T}}^{d}_{n}}}}{\bar{\eta }}_x ({\bar{\eta }}_{x+e_i} -{\bar{\eta }}_{x+e_i}^\ell ) g_x^i \\&\quad = \sum _{i=1}^d {\sum _{x \in {{\mathbb {T}}^{d}_{n}}}}\;\; {{\!\!\sum _{\begin{array}{c} y, z \in {{\mathbb {T}}^{d}_{n}}\\ y \sim z \end{array}}\!\!}}\phi ^\ell (y,z) ({\bar{\eta }}_{x+y+e_i}-{\bar{\eta }}_{x+z+e_i}) {\bar{\eta }}_x g_x^i\\&\quad = {{\!\!\sum _{\begin{array}{c} y, z \in {{\mathbb {T}}^{d}_{n}}\\ y \sim z \end{array}}\!\!}}\biggl (\sum _{i=1}^d {\sum _{x \in {{\mathbb {T}}^{d}_{n}}}}\phi ^\ell (y-x,z-x) {\bar{\eta }}_{x-e_i} g_{x-e_i}^i \biggr ) ({\bar{\eta }}_y-{\bar{\eta }}_z)\\&\quad = {{\!\!\sum _{\begin{array}{c} y, z \in {{\mathbb {T}}^{d}_{n}}\\ y \sim z \end{array}}\!\!}}h_{y,z}^\ell (g;\eta ) ({\bar{\eta }}_y- {\bar{\eta }}_z), \end{aligned}$$

where $h_{y,z}^\ell (g): {\Omega _n}\rightarrow {\mathbb {R}}$ is defined as

$$\begin{aligned} h_{y,z}^\ell (g;\eta ):= \sum _{i=1}^d {\sum _{x \in {{\mathbb {T}}^{d}_{n}}}}\phi ^\ell (y-x,z-x) {\bar{\eta }}_{x-e_i} g_{x-e_i}^i \end{aligned}$$

for every $\eta \in {\Omega _n}$. Observe that $h_{y,z}^\ell (g)$ does not depend on $\eta _y$, $\eta _z$. From Lemma E.2 of [18], we have that

$$\begin{aligned} \int h_{y,z}^\ell (g) ({\bar{\eta }}_y- {\bar{\eta }}_z) f d{\nu _{\!\rho _*}^n}\le \beta _0 \int (\nabla _{y,z} \sqrt{f})^2 d {\nu _{\!\rho _*}^n}+ \frac{1}{\beta _0} \int h_{y,z}^\ell (g)^2 f d {\nu _{\!\rho _*}^n}\end{aligned}$$

for every $\beta _0 > 0$. Choosing $\beta _0 = \beta n^2$ and taking the sum over $y \sim z$, we see that

3.7 $$\begin{aligned} \int (V(g)-V^\ell (g)) f d {\nu _{\!\rho _*}^n}\le \beta \int { \Gamma _n^{\textrm{ex}} (\sqrt{f})} d {\nu _{\!\rho _*}^n}+ \frac{1}{2\beta n^2} \int {{\!\!\sum _{\begin{array}{c} y, z \in {{\mathbb {T}}^{d}_{n}}\\ y \sim z \end{array}}\!\!}}h_{y,z}^\ell (g)^2 f d {\nu _{\!\rho _*}^n}. \end{aligned}$$

This estimate is exactly Lemma 3.3 of [18]. In our case, the proof is simpler due to the translation invariance of the reference measure ${\nu _{\!\rho _*}^n}$. Our next task is to take advantage of the average over boxes of size $\ell$ in $V^\ell (g)$ in order to get a good estimate in terms of $H(f; {\nu _{\!\rho _*}^n})$. The so-called *entropy inequality* says that for every function $h: \Omega _n \rightarrow {\mathbb {R}}$, every density *f* with respect to ${\nu _{\!\rho _*}^n}$ and every $\gamma > 0$,

3.8 $$\begin{aligned} \int h f d {\nu _{\!\rho _*}^n}\le \frac{1}{\gamma }\Big ( H(f; {\nu _{\!\rho _*}^n}) + \log \int e^{\gamma h} d {\nu _{\!\rho _*}^n}\Big ). \end{aligned}$$

In order to be able to use this estimate, we need to compute the exponential moments of the variables $h_{x,y}^\ell (g)^2$ and ${\overset{\leftarrow }{\eta }}_{x}^{\ell ,i}(g) {\overset{\rightarrow }{\eta }}_{x+e_i}^{\ell }$ with respect to ${\nu _{\!\rho _*}^n}$. Using independence and Lemma A.1, we see that for every $\theta \in {\mathbb {R}}$,

3.9 $$\begin{aligned} \log \int e^{\theta h_{y,z}^\ell (g)} d {\nu _{\!\rho _*}^n}&\le {\sum _{x \in {{\mathbb {T}}^{d}_{n}}}}\frac{\theta ^2}{8} \Big ( \sum _{i=1}^d \phi ^\ell (y-x-e_i,z-x-e_i) g_x^i \Big )^2 \nonumber \\&\le \frac{d \theta ^2}{8} \Vert g\Vert _\infty ^2 \sum _{i=1}^d {\sum _{x \in {{\mathbb {T}}^{d}_{n}}}}\phi ^\ell (y-x-e_i,z-x-e_i)^2\nonumber \\&\le C(d) \theta ^2 \Vert g\Vert _\infty ^2 g_d(\ell ). \end{aligned}$$

Therefore, by Lemma A.2,

3.10 $$\begin{aligned} \int e^{\gamma h_{y,z}^\ell (g)^2} d {\nu _{\!\rho _*}^n}\le \big (1- C(d) \gamma \Vert g\Vert _\infty ^2 g_d(\ell )\big )^{-1/2} \end{aligned}$$

whenever $$\gamma ^{-1} > C(d) \Vert g\Vert _\infty ^2 g_d(\ell )$$.

Observe that $h_{y,z}^\ell (g)$ and $h_{y',z'}^\ell (g)$ are independent whenever $|y+z-y'-z'| > 4 \ell$. Therefore, the set ${\mathcal {E}}_n^d$ can be divided into $C(d) \ell ^d$ disjoint sets $\{A^i; i \in {\mathcal {I}}\}$ such that the terms in the sums

$$\begin{aligned} \sum _{(y,z) \in A^i} h_{y,z}^\ell (g)^2 \end{aligned}$$

are mutually independent. Therefore, for $\gamma > 0$ small enough,

$$\begin{aligned} \int {{\!\!\sum _{\begin{array}{c} y, z \in {{\mathbb {T}}^{d}_{n}}\\ y \sim z \end{array}}\!\!}}h_{y,z}^\ell (g) ^2 f d {\nu _{\!\rho _*}^n}&= \sum _{i \in {\mathcal {I}}} \int \sum _{(y,z) \in A^i} h_{y,z}^\ell (g)^2 f d {\nu _{\!\rho _*}^n}\\&\le \sum _{i \in {\mathcal {I}}} \frac{1}{\gamma } \Big ( H(f; {\nu _{\!\rho _*}^n}) + \log \int e^{\gamma \sum _{(y,z) \in A^i} h_{y,z}^\ell (g)^2 } d {\nu _{\!\rho _*}^n}\Big )\\&\le \sum _{i \in {\mathcal {I}}} \frac{1}{\gamma } \Big (H(f; {\nu _{\!\rho _*}^n}) +\sum _{(y,z) \in A^i}\log \int e^{\gamma h_{y,z}^\ell (g)^2 } d {\nu _{\!\rho _*}^n}\Big )\\&\le \gamma ^{-1}C(d) \ell ^d H(f; {\nu _{\!\rho _*}^n}) +\gamma ^{-1} n^d \log \big (1- \gamma C(d) \Vert g\Vert _\infty ^2 g_d(\ell ) \big )^{-1/2}. \end{aligned}$$

In the last line we used (3.10). Taking $\gamma ^{-1}=2 C(d) \Vert g\Vert _\infty ^2 g_d(\ell )$ we conclude that

3.11 $$\begin{aligned} \int {{\!\!\sum _{\begin{array}{c} y, z \in {{\mathbb {T}}^{d}_{n}}\\ y \sim z \end{array}}\!\!}}h_{y,z}^\ell (g)^2 f d {\nu _{\!\rho _*}^n}\le C(d) \Vert g\Vert _\infty ^2 \ell ^d g_d(\ell ) \Big ( H(f; {\nu _{\!\rho _*}^n}) + \frac{n^d}{\ell ^d}\Big ). \end{aligned}$$

Repeating the arguments in (3.9) for the random variables ${\overset{\leftarrow }{\eta }}_{x}^{\ell ,i}(g)$ and ${\overset{\rightarrow }{\eta }}_{x}^{\ell }$ for each $x \in {{\mathbb {T}}^{d}_{n}}$ and each $i \in \{1,\dots ,d\}$, we see that for every $\theta \in {\mathbb {R}}$,

$$\begin{aligned} \log \int e^{\theta {\overset{\rightarrow }{\eta }}_{x}^{\ell }} d {\nu _{\!\rho _*}^n}\le \sum _{y \in {{\mathbb {T}}^{d}_{n}}} \tfrac{1}{8} p^\ell (y)^2 \theta ^2 \le \frac{\theta ^2}{8 \ell ^d} \end{aligned}$$

and

$$\begin{aligned} \log \int e^{\theta {\overset{\leftarrow }{\eta }}_{x}^{\ell ,i}(g)} d {\nu _{\!\rho _*}^n}\le \frac{\Vert g\Vert _\infty ^2 \theta ^2}{8 \ell ^d}. \end{aligned}$$

Since ${\overset{\leftarrow }{\eta }}_{x}^{\ell ,i}(g)$ and ${\overset{\rightarrow }{\eta }}_{x+e_i}^{\ell }$ are independent under ${\nu _{\!\rho _*}^n}$, using Lemma A.2 we obtain the bound

$$\begin{aligned} \int e^{\gamma {\overset{\leftarrow }{\eta }}_{x}^{\ell ,i}(g) {\overset{\rightarrow }{\eta }}_{x+e_i}^{\ell }} d {\nu _{\!\rho _*}^n}\le \int \exp \Big \{\frac{\Vert g\Vert _\infty ^2 \gamma ^2}{8 \ell ^d}\big ( {\overset{\rightarrow }{\eta }}_{x+e_i}^{\ell }\big )^2\Big \} d {\nu _{\!\rho _*}^n}\le \Big (1 - \frac{\gamma ^2 \Vert g\Vert _\infty ^2}{16 \ell ^{2d}}\Big )^{-1/2} \end{aligned}$$

for every $\gamma < 4 \ell ^d \Vert g\Vert _\infty ^{-1}$. Observe that for $\gamma =\frac{2 \ell ^d}{\Vert g\Vert _\infty }$, we obtain the bound

$$\begin{aligned} \int e^{\gamma {\overset{\leftarrow }{\eta }}_{x}^{\ell ,i}(g) {\overset{\rightarrow }{\eta }}_{x+e_i}^{\ell }} d {\nu _{\!\rho _*}^n}\le \frac{2}{\sqrt{3}}. \end{aligned}$$

Observe that the random variables ${\overset{\leftarrow }{\eta }}_{x}^{\ell ,i}(g) {\overset{\rightarrow }{\eta }}_{x+e_i}^{\ell }$ and ${\overset{\leftarrow }{\eta }}_{y}^{\ell ,j}(g) {\overset{\rightarrow }{\eta }}_{y+e_j}^{\ell }$ are independent under ${\nu _{\!\rho _*}^n}$ as soon as $\Vert y-x\Vert > 2\ell$. Dividing the set ${\mathcal {E}}_n^d$ as above, we obtain the estimate

3.12 $$\begin{aligned} \int {\sum _{x \in {{\mathbb {T}}^{d}_{n}}}}\sum _{i=1}^d {\overset{\leftarrow }{\eta }}_{x}^{\ell ,i}(g) {\overset{\rightarrow }{\eta }}_{x+e_i}^{\ell } f d {\nu _{\!\rho _*}^n}\le C(d) \Vert g\Vert _\infty \Big ( H(f; {\nu _{\!\rho _*}^n}) + \frac{n^d}{\ell ^d}\Big ). \end{aligned}$$

Putting estimates (3.7), (3.11) and (3.12) together, we conclude that for every $\beta >0$,

3.13 $$\begin{aligned} \int {\sum _{x \in {{\mathbb {T}}^{d}_{n}}}}\sum _{i=1}^d {\bar{\eta }}_x {\bar{\eta }}_{x+e_i} g_x^i f d {\nu _{\!\rho _*}^n}&\le \beta \int { \Gamma _n^{\textrm{ex}} (\sqrt{f})} d {\nu _{\!\rho _*}^n}\nonumber \\&\quad + C(d) \Big (\Vert g\Vert _\infty + \frac{\Vert g\Vert _\infty ^2 \ell ^d g_d(\ell )}{\beta n^2}\Big ) \Big ( H(f; {\nu _{\!\rho _*}^n}) + \frac{n^d}{\ell ^d}\Big ). \end{aligned}$$

Choosing in this estimate $\beta = \frac{1}{4}$ and

$$\begin{aligned} \ell =\left\{ \begin{array}{ccl} \frac{n}{4} &{}; &{} d=1,\\ \frac{n}{\sqrt{\log n}} &{}; &{} d=2,\\ n^{d-2} &{}; &{} d \ge 3, \end{array}\right. \end{aligned}$$

we see that

3.14 $$\begin{aligned} \int {\sum _{x \in {{\mathbb {T}}^{d}_{n}}}}\sum _{i=1}^d {\bar{\eta }}_x {\bar{\eta }}_{x+e_i} g_x^i f d {\nu _{\!\rho _*}^n}&\le \frac{1}{4} \int { \Gamma _n^{\textrm{ex}} (\sqrt{f})} d {\nu _{\!\rho _*}^n}+C(d) {\mathcal {A}}(\Vert g\Vert _\infty ) \nonumber \\&\big ( H(f; {\nu _{\!\rho _*}^n}) + n^{d-2} g_d(n)\big ). \end{aligned}$$

By the definition in (3.4) and Lemma 3.1,

$$\begin{aligned} H(f; {\nu _{\!\rho _*}^n}) \le \kappa ({\rho _*}) \int { \Gamma _n^{\textrm{r}} (\sqrt{f})} d {\nu _{\!\rho _*}^n}\end{aligned}$$

for every density *f*. Therefore,

$$\begin{aligned} \int {\sum _{x \in {{\mathbb {T}}^{d}_{n}}}}\sum _{i=1}^d {\bar{\eta }}_x {\bar{\eta }}_{x+e_i} g_x^i f d {\nu _{\!\rho _*}^n}&\le \frac{1}{4} \int {\Gamma _n^{\textrm{ex}} (\sqrt{f})} d {\nu _{\!\rho _*}^n}+ C \kappa ({\rho _*}) {\mathcal {A}}(\Vert g\Vert _\infty ) \\&\quad \times \int { \Gamma _n^{\textrm{r}} (\sqrt{f})} d {\nu _{\!\rho _*}^n}+ C {\mathcal {A}}(\Vert g\Vert _\infty ) n^{d-2} g_d(n), \end{aligned}$$

which proves the lemma. $\square$

### Proof of Theorem 2.4

Using Theorem 3.3, it is not difficult to carry out the proof of Theorem 2.4. If we take $g_x^ i = \frac{\lambda }{2 d {\rho _*}}$ for every $x \in {{\mathbb {T}}^{d}_{n}}$ and every $i \in \{1,\dots ,d\}$, if we take $f = f_t^ n$ in Theorem 3.3 and if we put the resulting estimate into (3.2), then we see that

$$\begin{aligned} H_n' (t)\le & {} - \frac{3}{4} \int {\Gamma _n^{\textrm{ex}} \left( \sqrt{f_t^n}\right) } d {\nu _{\!\rho _*}^n}+ \big ( C \kappa ({\rho _*}) {\mathcal {A}} \Big ( \frac{|\lambda |}{d {\rho _*}}\Big ) - 1 \big ) \times \\{} & {} \times \int {\Gamma _n^{\textrm{r}} \left( \sqrt{f_t^ n}\right) } d {\nu _{\!\rho _*}^n}+ C {\mathcal {A}}\Big ( \frac{|\lambda |}{d {\rho _*}} \Big ) n^{d-2} g_d(n). \end{aligned}$$

Observe that

$$\begin{aligned} \lim _{\lambda \rightarrow 0} \varepsilon _0 = \min \{a,b\}, \; \lim _{\lambda \rightarrow 0} {\rho _*}= \frac{a}{a+b} \text { and } \lim _{\lambda \rightarrow 0} \kappa ({\rho _*}) = \frac{|b^ 2 - a^ 2| \min \{a,b\}}{2 ab |\log \frac{b}{a}|}. \end{aligned}$$

Therefore,

$$\begin{aligned} \lim _{\lambda \rightarrow 0} \left( C\kappa ({\rho _*}) {\mathcal {A}} \Big (\frac{|\lambda |}{d {\rho _*}} \Big ) -1 \right) =-1 \end{aligned}$$

and there exists $\lambda _c>0$ such that

3.15 $$\begin{aligned} C \kappa ({\rho _*}) {\mathcal {A}}\Big ( \frac{\lambda }{d {\rho _*}}\Big ) < \frac{1}{2} \text { and } {\mathcal {A}} \Big ( \frac{\lambda }{d {\rho _*}} \Big ) \le 1 \end{aligned}$$

for every $\lambda \in [-\lambda _c,\lambda _c]$. Using Lemma 3.1, we see that for $\lambda \in [-\lambda _c,\lambda _c]$,

$$\begin{aligned} H_n'(t)&\le - \frac{1}{2} \int { \Gamma _n^{\textrm{r}} (\sqrt{f_t^ n})} d {\nu _{\!\rho _*}^n}+ C {\mathcal {A}} \Big ( \frac{|\lambda |}{d {\rho _*}} \Big ) n^{d-2} g_d(n)\\&\le -\frac{1}{2 \kappa ({\rho _*})} H_n(t) + C n^{d-2} g_d(n). \end{aligned}$$

Using $e^{\frac{t}{2 \kappa ({\rho _*})}}$ as an integrating factor, since $H_n(0) =0$ we conclude that

$$\begin{aligned} H( f_t^n; {\nu _{\!\rho _*}^n}) = H_n(t) \le C n^{d-2} g_d(n) \end{aligned}$$

for every $t \ge 0$. Taking $t \rightarrow \infty$, Theorem 2.4 is proved.

### Quantitative hydrostatics

In this section we prove Theorem 2.3 as a corollary of Theorem 2.4. It will be useful to prove a more general version of Theorem 2.3. Recall the definitions introduced before Theorem 2.6. We say that a function $\psi : \Omega _n \rightarrow {\mathbb {R}}$ has *support in* $B_R$ if $\psi (\eta ^x) = \psi (\eta )$ for every $x \notin {\mathcal {P}}(B_R)$ and every $\eta \in \Omega _n$. For $x \in {{\mathbb {T}}^{d}_{n}}$ and $\eta \in \Omega _n$, let $\tau _x \eta \in \Omega _n$ be the translation of $\eta$ by *x*, that is, $(\tau _x \eta )_z = \eta _{z+x}$ for every $z \in {{\mathbb {T}}^{d}_{n}}$. For $g: \Omega _n \rightarrow {\mathbb {R}}$ and $x \in {{\mathbb {T}}^{d}_{n}}$, let $\tau _x g: \Omega _n \rightarrow {\mathbb {R}}$ be the translation of *g* by *x*, that is, $\tau _x g(\eta ):= g(\tau _x \eta )$ for every $\eta \in \Omega _n$. For $\psi$ with support in $B_R$ and $x \in {{\mathbb {T}}^{d}_{n}}$, let us write $\psi _x: = \tau _x \psi$. Define $\langle \psi \rangle _{\rho _*}:= \int \psi d {\nu _{\!\rho _*}^n}$. For each $f \in {\mathcal {C}}({\mathbb {T}}^d; {\mathbb {R}})$, let $\Psi ^n(f): \Omega _n \rightarrow {\mathbb {R}}$ be given by

3.16 $$\begin{aligned} \Psi ^n(f; \eta ) := \frac{1}{n^d} \sum _{x \in {{\mathbb {T}}^{d}_{n}}} \big ( \psi _x(\eta ) - \langle \psi \rangle _{\rho _*}\big ) f \big ( \tfrac{x}{n} \big ) \end{aligned}$$

for every $\eta \in \Omega _n$. Theorem 2.3 is a particular case of the following estimate:

#### Theorem 3.4

There exists a finite constant $C = C(\psi ; {\rho _*})$ such that

$$\begin{aligned} \int \Psi ^n(f)^2 d {\mu _{\textrm{ss}}^n}\le \frac{C \Vert f\Vert _{\ell ^2_n}^2 g_d(n)}{n^2} \end{aligned}$$

for every $f \in {\mathcal {C}}({\mathbb {T}}^d; {\mathbb {R}})$, where

$$\begin{aligned} \Vert f\Vert _{\ell ^2_n}^2:= \frac{1}{n^d} \sum _{x \in {{\mathbb {T}}^{d}_{n}}} f \big ( \tfrac{x}{n} \big )^2. \end{aligned}$$

#### Proof

Let ${{\,\textrm{Osc}\,}}(\psi ):= \sup _\eta \psi (\eta ) - \inf _\eta \psi (\eta )$. By Hoeffding’s Lemma (see Lemma ()), for every $x \in {{\mathbb {T}}^{d}_{n}}$ we have that

$$\begin{aligned} \log \int e^{ \theta ( \psi _x -\langle \psi \rangle _{\rho _*})} d {\nu _{\!\rho _*}^n}\le \frac{\theta ^2 {{\,\textrm{Osc}\,}}(\psi )^2}{8}. \end{aligned}$$

Since $B_R$ is a cube of side $2R+1$, $\psi _x$ and $\psi _y$ are independent as soon as $\Vert y-x \Vert _\infty > 2R+1$. Therefore, ${{\mathbb {T}}^{d}_{n}}$ can be split into disjoint sets $(A^i; i \in {\mathcal {I}}_R)$ such that the cardinality of ${\mathcal {I}}_R$ is at most $C(d)(2R+1)^d$ and such that the terms in the sums

$$\begin{aligned} \frac{1}{n^d} \sum _{x \in A^i} \big (\psi _x - \langle \psi \rangle _{{\rho _*}}\big ) f \big (\tfrac{x}{n} \big ) \end{aligned}$$

are all independent. Therefore,

$$\begin{aligned} \log \!\int \! e^{\theta \Psi ^n(f) } d {\nu _{\!\rho _*}^n}&\!\le \! \frac{C(d)}{(2R+1)^d} \!\sum _{i \in {\mathcal {I}}_R} \log \int \exp \big \{ \frac{\theta C(d)(2R\!+\!1)^d}{ n^{d}} \!\sum _{x \in A^i} (\psi _x\!-\! \langle \psi \rangle _{\rho _*}) f \big ( \frac{x}{n} \big )\big \} d {\nu _{\!\rho _*}^n}\\&\le \frac{C(d)}{(2R+1)^d} \sum _{i \in {\mathcal {I}}_R} \sum _{x \in A^i} \frac{\theta ^2 C(d) (2R+1)^{2d} {{\,\textrm{Osc}\,}}(\psi )^2}{8 n^{2d}} f\big ( \frac{x}{n} \big )^2 \\&\le \frac{\theta ^2 C(d) (2R+1)^d {{\,\textrm{Osc}\,}}(\psi )^2 \Vert f\Vert _{\ell ^2_n}^2}{n^d}. \end{aligned}$$

By Lemma A.2 we see that

$$\begin{aligned} \int e^{\theta \Psi ^ n(f)^2} d {\nu _{\!\rho _*}^n}\le \sqrt{2} \end{aligned}$$

for

$$\begin{aligned} \theta = \frac{8 n^d}{C(d)(2R+1)^d \Vert f\Vert _{\ell ^2_n}^2{{\,\textrm{Osc}\,}}(\psi )^2}. \end{aligned}$$

Using the entropy inequality (3.8), we see that

3.17 $$\begin{aligned} \int \Psi ^ n(f)^ 2 d {\mu _{\textrm{ss}}^n}&\le \theta ^{-1} \Big ( H({\mu _{\textrm{ss}}^n}| {\nu _{\!\rho _*}^n}) + \log \int e^{\theta \Psi ^ n(f)^2} d {\nu _{\!\rho _*}^n}\Big )\nonumber \\&\le \frac{C(d)(2R+1)^d \Vert f\Vert _{\ell ^2_n}^2 {{\,\textrm{Osc}\,}}(\psi )^2}{n^d} \big ( C n^{d-2} g_d(n) + \log \sqrt{2} \big )\nonumber \\&\le \frac{C(\psi , {\rho _*},d) \Vert f\Vert _{\ell ^2_n}^2 g_d(n)}{n^2}, \end{aligned}$$

as we wanted to show. $\square$

Observe that Theorem 2.3 corresponds to Theorem 3.4 in the particular case $\psi (\eta ) = \eta _0$. It turns out that Theorem 3.4 can also be used to prove Theorem 2.6.

#### Proof of Theorem 2.6

In general, the bound on the relative entropy given by Theorem 2.4 is not enough to derive a result as strong as Theorem 2.6. However, in our particular setting, we can take advantage of the translation invariance of the dynamics in order to improve the bounds. Observe that the generator of $(\eta (t); t \ge 0)$ satisfies

$$\begin{aligned} \tau _x (L_nf) = L_n (\tau _x f) \end{aligned}$$

for every $f: \Omega _n \rightarrow {\mathbb {R}}$ and every $x \in {{\mathbb {T}}^{d}_{n}}$. In particular, if the law of $\eta (0)$ is translation invariant, then the law of $\eta (t)$ is translation invariant for every $t \ge 0$. Taking $t \rightarrow \infty$, we deduce that ${\mu _{\textrm{ss}}^n}$ is translation invariant.

Recall the definition of $\Pi _R$ given before Theorem 2.6. For $R <\frac{n-1}{2}$ and $x \in {{\mathbb {T}}^{d}_{n}}$, let $\Pi _R^x$ be the canonical projection onto the box of size *R* and center *x*, that is, $\Pi _R^x \eta := \Pi _R( \tau _x \eta )$ for every $\eta \in \Omega _n$. Observe that for every $\psi : \{0,1\}^{B_R} \rightarrow {\mathbb {R}}$ and every $x,y \in {{\mathbb {T}}^{d}_{n}}$,

$$\begin{aligned} \int \psi (\Pi _R^x \eta ) d {\mu _{\textrm{ss}}^n}= \int \psi (\Pi _R^y \eta ) d {\mu _{\textrm{ss}}^n}. \end{aligned}$$

Therefore,

$$\begin{aligned} \int \psi \,d {\mu _{\textrm{ss}}^{n,R}} = \int \psi (\Pi _R^x \eta ) d {\mu _{\textrm{ss}}^n}\end{aligned}$$

for every $x \in {{\mathbb {T}}^{d}_{n}}$. Recall the definition of $\Psi ^n$ given in (3.16). Applying estimate (3.17) to the constant function $\textbf{1}$, we see that

$$\begin{aligned} \Big | \int \psi \, d {\mu _{\textrm{ss}}^{n,R}} - \int \psi \, d {\nu _{\rho _*}^{n, R}} \Big |^2&= \Big | \int \frac{1}{n^ d} \sum _{x \in {{\mathbb {T}}^{d}_{n}}} \big ( \psi _x - \langle \psi \rangle _{\rho _*}) d {\mu _{\textrm{ss}}^n}\Big |^2\\&= \Big | \int \Psi ^n(\textbf{1}) d {\mu _{\textrm{ss}}^n}\Big |^2 \le \frac{C (2R+1)^d g_d(n){{\,\textrm{Osc}\,}}(\psi )^ 2}{n^2}. \end{aligned}$$

Observe that $\Vert \psi \Vert _\infty \le 1$ implies ${{\,\textrm{Osc}\,}}(\psi ) \le 2$. Taking the supremum over $\Vert \psi \Vert _\infty \le 1$ in this estimate and recalling the definition of $g_d(n)$, Theorem 2.6 is proved. $\square$

## Density fluctuations

### Case $d=1$

In this section we prove Theorem 2.7 in the case $d=1$. In that case the proof is simpler, due to the fact that the estimate in Theorem 2.4 is uniform in *n*. This will allow us to explain better the ideas behind the proof, which will be used for $d=2,3$ afterwards. Some of the computations are not dimension dependent; in those cases we will keep the dependence on *d* in the notation.

By Theorem 2.3, for every $\lambda \in [-\lambda _c,\lambda _c]$,

$$\begin{aligned} \int X^n(f)^2 d {\mu _{\textrm{ss}}^n}\le C \Vert f\Vert _{\ell ^2_n}^2. \end{aligned}$$

Recall definition (2.8). For $f(x) = \cos (2 \pi k x)$ or $f = \sin (2 \pi k x)$, $\Vert f\Vert _{\ell ^2_n} \le 1$. Therefore, for $m < -1/2$,

$$\begin{aligned} \int \Vert X^n\Vert _{{\mathcal {H}}^m}^2 d {\mu _{\textrm{ss}}^n}= \sum _{k \in {\mathbb {Z}}} (1+k^2)^m \int |\hat{X}^n(k)|^2 d {\mu _{\textrm{ss}}^n}\le C \sum _{k \in {\mathbb {Z}}} (1+k^2)^m \le C(m) <+\infty . \end{aligned}$$

Recall that the inclusion ${\mathcal {H}}^{m} \subseteq {\mathcal {H}}^{m'}$ is compact if $m' < m$, and therefore balls in ${\mathcal {H}}^{-m'}$ are compact in ${\mathcal {H}}^{-m}$. Therefore, for $m' \in (m,-1/2)$ the set $\{ \Vert X\Vert _{{\mathcal {H}}^{m'}} \le M\}$ is compact in ${\mathcal {H}}^m$. From the previous estimate we see that

$$\begin{aligned} {\mu _{\textrm{ss}}^n}\big ( \Vert X^n\Vert _{{\mathcal {H}}^{m'}} > M\big ) \le \frac{C(m')}{M^2}. \end{aligned}$$

Taking $M \rightarrow \infty$, we conclude that the sequence $\{X^n; n \in {\mathbb {N}}\}$ is tight in ${\mathcal {H}}^{m}$.

The idea is to prove a limit theorem for the *dynamical* fluctuation field, and to derive Theorem 2.7 from such result. Let $(X^n_t; t \ge 0)$ be the process given by

4.1 $$\begin{aligned} X_t^n(f) := X^n(\eta ^n(t),f) = \frac{1}{n^ {d/2}} \sum _{x \in {{\mathbb {T}}^{d}_{n}}} (\eta _x^n(t) - {\rho _*}) f \big ( \tfrac{x}{n} \big ) \end{aligned}$$

for every $f \in {\mathcal {C}}^\infty ({\mathbb {T}}^d; {\mathbb {R}})$. The following result is a version of [17, Theorem 2.1] for the model considered here, and it holds for dimensions $d \le 3$:

#### Proposition 4.1

Let $(\mu _0^n; n \in {\mathbb {N}})$ be a sequence of probability measures on $\Omega _n$ such that:

(i) $$\displaystyle {\sup _{n \in {\mathbb {N}}} H(\tfrac{d \mu _0^n}{d {\nu _{\!\rho _*}^n}}; {\nu _{\!\rho _*}^n}) <+\infty }$$,
(ii) $X^n \rightarrow X_0$ in ${\mathcal {H}}^{-m}$ in law with respect to $\mu _0^n$ for some $m > d/2$.

For $d \le 3$, the sequence $(X_t^n; t \ge 0)_{n \in {\mathbb {N}}}$ converges in the sense of finite-dimensional distributions to the process $(X_t; t \ge 0)$, solution of the equation

4.2 $$\begin{aligned} \partial _t X_t = \Delta X_t + F' ({\rho _*}) X_t + \sqrt{2\chi ({\rho _*})} \nabla \cdot \dot{{\mathcal {W}}}^1 + \sqrt{G({\rho _*})} \dot{{\mathcal {W}}}^2, \end{aligned}$$

with initial condition $X_0$, where $\dot{{\mathcal {W}}}^1$ is an ${\mathbb {R}}^d$-valued white noise, $\dot{{\mathcal {W}}}^2$ is a real-valued white noise and $\dot{{\mathcal {W}}}^i$, $i=1,2$ are independent.

We start proving a lemma:

#### Lemma 4.2

For every dimension $d \ge 1$ and every $m > d/2$, the Eq. (4.2) has a unique stationary solution supported in ${\mathcal {H}}^{-m}$.

#### Proof

Let $(P_t; t \ge 0)$ be the semigroup generated by the operator $\Delta +F'({\rho _*})$. For every $f \in {\mathcal {C}}^\infty ({\mathbb {T}}^d; {\mathbb {R}})$ and every $t \ge 0$,

4.3 $$\begin{aligned} X_t(f) = X_0(P_t f) + \int _0^t \sqrt{2 \chi ({\rho _*})} d {\mathcal {W}}_s^1(P_{t-s} \nabla f) + \int _0^t \sqrt{G({\rho _*})} d {\mathcal {W}}_s^2(P_{t-s} f). \end{aligned}$$

Since $F'({\rho _*})<0$, we see that $P_t f \rightarrow 0$ as $t \rightarrow \infty$ exponentially fast in ${\mathcal {H}}^m$ for every $m \in {\mathbb {R}}$. In consequence, the random family $(X_\infty (f); f \in {\mathcal {C}}^\infty ({\mathbb {T}}^d; {\mathbb {R}}))$ given by

$$\begin{aligned} X_\infty (f):= \lim _{t \rightarrow \infty } X_t(f) \end{aligned}$$

for every $f \in {\mathcal {C}}^\infty ({\mathbb {T}}^d; {\mathbb {R}})$ is well defined in law. Moreover, $X_\infty$ is a well-defined ${\mathcal {H}}^{-m}$-valued random variable for $m>d/2$, and it satisfies the identity

4.4 $$\begin{aligned} X_\infty (f) = \int _0^\infty \Big ( \sqrt{2 \chi ({\rho _*})} d {\mathcal {W}}_t^1(P_{t} \nabla f) +\sqrt{G({\rho _*})} d {\mathcal {W}}_t^2(P_{t} f)\Big ), \end{aligned}$$

where this identity is understood as an identity in law for ${\mathcal {H}}^{-m}$-valued random variables. We claim that $X_\infty$ is the unique stationary state of (4.2). In fact, if there is another stationary state $Y_\infty$, the solution of (4.2) with initial condition $Y_\infty$ on one hand satisfies $X_t = Y_\infty$ in law for every $t \ge 0$ and on the other hand it converges to $X_\infty$.

The right-hand side of (4.4) is a centered Gaussian process. Therefore, it is characterized by its covariance operator. Observe that

$$\begin{aligned} {\mathbb {E}}[X_\infty (f)^2] = 2 \chi ({\rho _*}) \int _0^\infty \Vert P_t \nabla f\Vert ^2_{ L^2} dt + G({\rho _*}) \int _0^\infty \Vert P_t f\Vert ^2_{ L^2} dt. \end{aligned}$$

In Fourier space,

$$\begin{aligned} \widehat{P_t f}(k)= e^{ - 4\pi ^2 \Vert k\Vert ^2 t + F'({\rho _*}) t} \hat{f}(k). \end{aligned}$$

Therefore, by Parseval’s identity,

4.5 $$\begin{aligned} {\mathbb {E}}[X_\infty (f)^2] = \sum _{k \in {\mathbb {Z}}^d} \big |\hat{f}(k)\big |^2 \Big ( \frac{4 \pi ^2 \Vert k\Vert ^2 \chi ({\rho _*})}{4 \pi ^2 \Vert k\Vert ^2-F'({\rho _*})} + \frac{G({\rho _*})}{8 \pi ^2 \Vert k\Vert ^2 -2 F'({\rho _*})}\Big ), \end{aligned}$$

which is equal to the expression given in (2.9) for the limit process *X*. Observe that

4.6 $$\begin{aligned} \lim _{\Vert k\Vert \rightarrow \infty } \Big ( \frac{4 \pi ^2 \Vert k\Vert ^2 \chi ({\rho _*})}{4 \pi ^2 \Vert k\Vert ^2-F'({\rho _*})} +\frac{G({\rho _*})}{8 \pi ^2 \Vert k\Vert ^2 -2 F'({\rho _*})}\Big ) = \chi ({\rho _*}), \end{aligned}$$

and in particular the coefficients in (4.5) are bounded in *k*. Therefore, ${\mathbb {E}}[\Vert X_\infty \Vert _{{\mathcal {H}}^{-m}}^2] <+\infty$ for every $m >d/2$ and in particular $X_\infty$ is a well-defined random variable in ${\mathcal {H}}^{-m}$ for every $m > d/2$.

Let us go back to the proof of Theorem 2.7. We have already proved that the sequence $(X^n; n \in {\mathbb {N}})$ is tight in ${\mathcal {H}}^{-m}$ with respect to ${\mu _{\textrm{ss}}^n}$, for $m > 1/2$. Let $n'$ be a subsequence for which $X^{n'}$ converges in law with respect to the topology of ${\mathcal {H}}^{-m}$ to a random variable $\widetilde{X}$. By Theorem 2.4, the measures ${\mu _{\textrm{ss}}^{n'}}$ satisfy the conditions of Proposition 4.1. Since ${\mu _{\textrm{ss}}^{n'}}$ is stationary, $\widetilde{X}$ is a stationary solution of (4.2). By Lemma 4.2, $\widetilde{X} = X_\infty$. We have just showed that $(X^n; n \in {\mathbb {N}})$ is relatively compact and it has a unique accumulation point $X_\infty$. We conclude that $X^n$ converges to $X_\infty$, as we wanted to show.

### Case $d=2,3$

In this section we prove Theorem 2.7 in the case $d=2,3$. In this case, the proof of Theorem 2.7 presented in the previous section does not work, because the entropy bound of Theorem 2.4 is not strong enough to derive tightness of the sequence $(X^n; n \in {\mathbb {N}})$ with respect to $({\mu _{\textrm{ss}}^n}; n \in {\mathbb {N}})$. The idea is to adapt the proof of Lemma 4.2, *directly* to the process $(X_t^n; t \ge 0)$ defined in (4.1). In order to do that, let us introduce the Dynkin’s martingales associated to $(X_t^n; t \ge 0)$. For every $T >0$ and every $g \in {\mathcal {C}}^{1,\infty }( [0,T] \times {\mathbb {T}}^d; {\mathbb {R}})$, the process $({\mathcal {M}}_t^n(g); t \in [0,T])$ given by

4.7 $$\begin{aligned} {\mathcal {M}}_t^n(g) := X_t^n(g_t) - X_0^n(g_0) - \int _0^t (\partial _s+L_n) X_s^n(g_s) ds \end{aligned}$$

is a martingale of predictable quadratic variation

4.8 $$\begin{aligned} \langle {\mathcal {M}}^n(g)\rangle _t = \int _0^t {\Gamma _n (X_s^n(g))} ds. \end{aligned}$$

Our aim is to choose $g_s$ in such a way that Eqs. (4.7), (4.8) are approximate versions of (4.3). After some explicit computations, we see that

4.9 $$\begin{aligned} (\partial _s+L_n) X^n(g_s) = X^n((\partial _s + {\mathbb {L}}_n)g_s) + \frac{1}{n^{d/2}} V( g_{s}) \end{aligned}$$

where the linear operator ${\mathbb {L}}_n$ is defined as

$$\begin{aligned} {\mathbb {L}}_n f (x):= \Big ( n^2 + \frac{\lambda (1-{\rho _*})}{2d} \Big ) \sum _{\begin{array}{c} y \in {\mathbb {T}}^d_n\\ y \sim 0 \end{array}} \big ( f \big ( x+\tfrac{y}{n} \big ) - f (x) \big ) + F'({\rho _*}) f (x) \end{aligned}$$

for every $f \in {\mathcal {C}}({\mathbb {T}}^d; {\mathbb {R}})$ and every $x \in {\mathbb {T}}^d$, where *V* was defined in (3.5) and where $g_{s}$ is defined as

$$\begin{aligned} g^i_{s}(x):= -\frac{\lambda }{2 d} \big ( g_{s} ( x ) + g_{s} \big (x + \tfrac{e_i}{n}\big )\big ) \end{aligned}$$

for every $i \in \{1,\dots ,d\}$ and every $x \in {\mathbb {T}}^d$. We also see that

4.10 $$\begin{aligned} \Gamma _n X^n(g_s) = \frac{1}{2 n^d} {\!\!\sum _{\begin{array}{c} x, y \in {{\mathbb {T}}^{d}_{n}}\\ x \sim y \end{array}}\!\!}(\eta _y - \eta _x)^2 n^2 \big ( g_s\big ( \tfrac{y}{n} \big ) - g_s\big ( \tfrac{x}{n} \big ) \big )^2 + \frac{1}{n^d} {\sum _{x \in {{\mathbb {T}}^{d}_{n}}}}c_x(\eta ) g_s\big ( \tfrac{x}{n} \big )^2. \end{aligned}$$

Observe that ${\mathbb {L}}_n$ is a discrete approximation of the operator ${\mathbb {L}}:= \Delta + F'({\rho _*})$ used in the proof of Lemma 4.2. For $f \in {\mathcal {C}}^\infty ({\mathbb {T}}^d; {\mathbb {R}})$ and $T >0$, let $(g_{t,T}; T >0, t \in [0,T])$ be given by $g_{t,T}:= P_{T-t} f$ for every $T >0$ and every $t \in [0,T]$, where $(P_t; t \ge 0)$ is the semigroup generated by $\Delta + F'({\rho _*})$. For $T>0$ and $t \in [0,T]$, let ${\mathcal {M}}_{t,T}^n(f):= {\mathcal {M}}_t^n(g_{\cdot ,T})$. Observe that $({\mathcal {M}}_{t,T}^n(f); t \in [0,T])$ is a martingale and observe that

4.11 $$\begin{aligned} X_T^n(f)&= X_0^n(g_{0,T}) + \int _0^T (\partial _t + L_n) X_t^n(g_{t,T}) dt + {\mathcal {M}}_{T,T}^n(f)\nonumber \\&= X_0^n(g_{0,T}) + \int _0^T X_t^n((\partial _t + {\mathbb {L}}_n) g_{t,T}) dt + I_T^n(V;f) + {\mathcal {M}}_{T,T}^n(f), \end{aligned}$$

where

$$\begin{aligned} I_T^n(V; f):= \int _0^T \frac{1}{n^{d/2}} V({g}_{t,T}) dt. \end{aligned}$$

Assuming that $X^n_0$ has law ${\mu _{\textrm{ss}}^n}$, this equation is the discrete version of (3.5) we are aiming for. The proof of Theorem 2.7 will follow from a careful analysis of equation (4.11). This analysis will be divided into three parts. In Sect. 4.3 we will derive various estimates that will then be used in Sect. 4.4 to prove tightness of the field $X^n$ and in Sect. 4.5 to prove convergence of finite-dimensional laws of $X^n$.

### Estimates

Our aim is to prove estimates in $L^2({\mathbb {P}}_{\mu _{\textrm{ss}}^n}^n)$ of each of the terms appearing in (4.11), except $I_T^n(V; f)$, which we can only estimate in $L^1({\mathbb {P}}_{\mu _{\textrm{ss}}^n}^n)$. Since $F' ({\rho _*})<0$, the semigroup $(P_t; t \ge 0)$ converges to 0 with respect to the uniform norm with exponential rate $-F' ({\rho _*})$. We start estimating the second moment of ${\mathbb {E}}_{{\mu _{\textrm{ss}}^n}}[X_0^n(g_{0,T})]$. From Theorem 2.3,

4.12 $$\begin{aligned} {\mathbb {E}}^n_{{\mu _{\textrm{ss}}^n}}[ X_0^n(g_{0,T} )^2]&\le \frac{C g_d(n)}{n^2} {\sum _{x \in {{\mathbb {T}}^{d}_{n}}}}P_T f \big (\tfrac{x}{n} \big )^2 \nonumber \\&\le C g_d(n) n^{d-2} \Vert f\Vert _\infty ^2 e^{2F'({\rho _*}) T}. \end{aligned}$$

In order to estimate the second moment of $\int _0^T X_t^n((\partial _t + {\mathbb {L}}_n) g_{t,T}) dt$, observe that $(\partial _t + {\mathbb {L}})g_{t,T}=0$. Therefore, by Taylor’s formula,

$$\begin{aligned}{}[ \big |(\partial _t + {\mathbb {L}}_n) g_{t,T} \big ( \tfrac{x}{n} \big ) \big | = \big |({\mathbb {L}}_n - {\mathbb {L}}) g_{t,T}\big ( \tfrac{x}{n} \big ) \big | \le \frac{1}{12 n^2} \big ( 6 \lambda (1-{\rho _*}) \Vert D^2 g_{t,T}\Vert _\infty +d\Vert D^4 g_{t,T}\Vert _\infty \big ). \end{aligned}$$

Since the differential operator *D* commutes with ${\mathbb {L}}$, we obtain the bound

$$\begin{aligned} \big |(\partial _t + {\mathbb {L}}_n) g_{t,T}\big ( \tfrac{x}{n} \big ) \big | \le \frac{C}{12 n^2} \big ( \Vert D^2 f\Vert _\infty +\Vert D^4f\Vert _\infty \big ) e^{F'({\rho _*})(T-t)}. \end{aligned}$$

Combining this bound with Theorem 2.3, the invariance of ${\mu _{\textrm{ss}}^n}$, and applying the Cauchy-Schwarz inequality in the time integral, we see that for every $\delta \in (0,-F' ({\rho _*}))$ and every $\lambda \in [-\lambda _c,\lambda _c]$,

4.13 $$\begin{aligned}&{\mathbb {E}}_{{\mu _{\textrm{ss}}^n}}^n \Big [\Big | \int _0^T X_t^n ((\partial _t + {\mathbb {L}}_n)g_{t,T}) dt \Big |^2\Big ]\nonumber \\&\quad \le \Big (\int _0^T e^{-2\delta t} dt \Big ) \Big ( \int _0^T e^{2 \delta t} {\mathbb {E}}_{{\mu _{\textrm{ss}}^n}}^n \big [ \big | X_t^n((\partial _t + {\mathbb {L}}_n) g_{t,T}) \big |^2 \big ] dt\Big )\nonumber \\&\quad \le \frac{C}{2 \delta } \int _0^T g_d(n) n^{d-6} (\Vert D^2 f\Vert _\infty ^2 + \Vert D^4 f\Vert _\infty ^2) e^{2(\delta +F'({\rho _*})) t} dt\nonumber \\&\quad \le C g_d(n) n^{d-6} (\Vert D^2 f\Vert _\infty ^2 + \Vert D^4 f\Vert _\infty ^2). \end{aligned}$$

The martingale term is easy to estimate. We have that

4.14 $$\begin{aligned} {\mathbb {E}}_{{\mu _{\textrm{ss}}^n}}^n[{\mathcal {M}}_{T,T}^n(f)^2]&= {\mathbb {E}}_{{\mu _{\textrm{ss}}^n}}^n \Big [ \int _0^T {\Gamma _n (X_t^n(g_{t,T}))}dt \Big ] \le \int _0^T C \big ( \Vert D g_{t,T}\Vert _\infty ^2 + \Vert g_{t,T}\Vert _\infty ^2\Big ) dt\nonumber \\&\le C\big ( \Vert D f\Vert _\infty ^2 + \Vert f\Vert _\infty ^2\Big ). \end{aligned}$$

Recall that due to the contractivity property of the semigroup $(P_t; t \ge 0)$, the constant *C* does not depend on *T*. The expectation ${\mathbb {E}}_{{\mu _{\textrm{ss}}^n}}^n [ | I_T^n(V; f) | ]$ is the most difficult to estimate. In fact, we are only able to obtain a first-moment estimate. By the entropy inequality (3.8) and $H( {\mathbb {P}}_{{\mu _{\textrm{ss}}^n}}^n | {\mathbb {P}}_{{\nu _{\!\rho _*}^n}}^n ) = H( {\mu _{\textrm{ss}}^n}| {\nu _{\!\rho _*}^n})$ (which is a consequence of Markov’s property) we have that

4.15 $$\begin{aligned} {\mathbb {E}}_{{\mu _{\textrm{ss}}^n}}^n [ | I_T^n(V; f) | ] \le \frac{1}{\theta } \Big ( H({\mu _{\textrm{ss}}^n}| {\nu _{\!\rho _*}^n}) \!+\! \log {\mathbb {E}}^n_{{\nu _{\!\rho _*}^n}} \big [ \exp \big \{ \theta | I_T^n(V; f) | \big \}\big ] \Big ). \end{aligned}$$

Using the elementary bounds $\exp |x|\le \exp (x)+\exp (-x)$, $\log (a+b) \le \log 2 + \max \{\log a, \log b\}$ and for the choice

$$\begin{aligned} \theta := \frac{\lambda _c n^{d/2}}{2 d {\rho _*}\sup _{t,T} \Vert g_{t,T}\Vert _\infty }, \end{aligned}$$

we see that

4.16 $$\begin{aligned} {\mathbb {E}}_{{\mu _{\textrm{ss}}^n}}^n [ | I_T^n(V; f) | ] \le \frac{2 d {\rho _*}\Vert g\Vert _\infty }{\lambda _c n^{d/2}} \Big ( H({\mu _{\textrm{ss}}^n}| {\nu _{\!\rho _*}^n}) + \log 2 + \max _{\pm } \log {\mathbb {E}}^n_{{\nu _{\!\rho _*}^n}} \big [ \exp \big \{ \pm \theta I_T^n(V; f) \big \}\big ] \Big ). \end{aligned}$$

To estimate the rightmost term in last display we use the following result.

#### Proposition 4.3

For every $T >0$ and every $W: [0,T] \times \Omega _n \rightarrow {\mathbb {R}}$,

$$\begin{aligned} \log {\mathbb {E}}^n_{{\nu _{\!\rho _*}^n}} \Big [ \exp \Big \{ \int _0^T W_t(\eta (t)) dt \Big \} \Big ] \le \int _0^T \sup _{f} \Big \{ \int \Big (W_t + \tfrac{1}{2} L_n^*\textbf{1} \Big ) f d {\nu _{\!\rho _*}^n}- \int { \Gamma _n (\sqrt{f})} d {\nu _{\!\rho _*}^n}\Big \}dt, \end{aligned}$$

where the supremum is taken over all densities *f* with respect to ${\nu _{\!\rho _*}^n}$.

This proposition corresponds to Lemma A.1.7.2 of [19] in the case in which the measure ${\nu _{\!\rho _*}^n}$ is reversible with respect to $L_n$ (and therefore $L_n^*\textbf{1} =0$). It was observed in [2, p.78] that this estimate is also valid if the measure ${\nu _{\!\rho _*}^n}$ is invariant but not reversible. A complete proof of Proposition 4.3 can be found in [24, Lemma 3.3] or [16, Lemma 9.1].

The idea is to take $$W_t = \theta n^{-d/2} V( g_{t,T})$$ in Proposition 4.3 and then to use Theorem 3.4 to turn the variational formula into a quantitative estimate. Observe that for $$W_t $$, by (3.3) we get

$$\begin{aligned} W_t + \frac{1}{2} L_n^*\textbf{1} = V \big ( \theta n^{-d/2} g_{t,T} + \frac{\lambda }{2 d {\rho _*}} \vec {1}), \end{aligned}$$

where $\vec {1}$ denotes the vector function equal to 1 in all coordinates. Therefore, by Theorem 3.3,

$$\begin{aligned}&\int \!\!\Big ( W_t + \frac{1}{2} L_n^*\textbf{1} \Big ) f d {\nu _{\!\rho _*}^n}\!\! -\!\! \int {\Gamma _n (\sqrt{f})} d {\nu _{\!\rho _*}^n}\\&\quad \le \Big ( C \kappa ({\rho _*}) {\mathcal {A}}\Big ( \frac{\theta }{n^{d/2}} \Vert g_{t,T}\Vert _\infty + \frac{\lambda }{2d {\rho _*}}\Big ) -1 \Big ) \int { \Gamma _n^r (\sqrt{f})} d {\nu _{\!\rho _*}^n}\\&\qquad + C {\mathcal {A}}\Big ( \frac{\theta }{n^{d/2}} \Vert g_{t,T}\Vert _\infty + \frac{\lambda }{2 d {\rho _*}}\Big ) g_d(n) n^{d-2}, \end{aligned}$$

for every $\lambda \in [-\lambda _c,\lambda _c]$, where the constant $\lambda _c$ was chosen in (3.15). From our choice of $\theta$ we conclude that

$$\begin{aligned} \int \Big ( W_t + \frac{1}{2} L_n^*\textbf{1} \Big ) f d {\nu _{\!\rho _*}^n}- \int {\Gamma _n (\sqrt{f})} d {\nu _{\!\rho _*}^n}\le C {\mathcal {A}} \Big ( \frac{\lambda _c}{d {\rho _*}}\Big ) g_d(n) n^{d-2}. \end{aligned}$$

From the right-hand side of (3.15) we obtain that

4.17 $$\begin{aligned} {\mathbb {E}}_{{\mu _{\textrm{ss}}^n}}^n [ | I_T^n(V; f) | ] \le C \Vert f \Vert _\infty g_d(n)n^{d/2 -2} (1+T). \end{aligned}$$

### Tightness

In this section we prove tightness of the sequence $(X^n; n \in {\mathbb {N}})$ with respect to the strong topology of ${\mathcal {H}}^{-m}$ for $m>d/2$. By Prohorov’s theorem, $(X^n; n \in {\mathbb {N}})$ is tight in ${\mathcal {H}}^{-m}$ if for every $\epsilon >0$ there exists a compact set $K_\epsilon$ in ${\mathcal {H}}^{-m}$ such that

$$\begin{aligned} {\mu _{\textrm{ss}}^n}( X^n \notin K_\epsilon ) \le \epsilon \end{aligned}$$

for every $n \in {\mathbb {N}}$. It can be shown that whenever $m < m'$, the embedding ${\mathcal {H}}^{-m} \subseteq {\mathcal {H}}^{-m'}$ is compact, which means that closed balls in ${\mathcal {H}}^{-m}$ are compact in ${\mathcal {H}}^{-m'}$. Therefore, in order to prove tightness of $(X^n; n \in {\mathbb {N}})$ in ${\mathcal {H}}^{-m'}$, it is enough to show that

$$\begin{aligned} \lim _{M \rightarrow \infty } \sup _{n \in {\mathbb {N}}} {\mu _{\textrm{ss}}^n}(\Vert X^n\Vert _{{\mathcal {H}}^{-m}} > M) =0 \end{aligned}$$

for some $m < m'$. Observe that for every $T >0$, the law of $X^n$ under ${\mu _{\textrm{ss}}^n}$ is equal to the law of $X_T^n$ under ${\mathbb {P}}_{\mu _{\textrm{ss}}^n}^n$. Therefore, it is enough to estimate the probabilities ${\mathbb {P}}_{\mu _{\textrm{ss}}^n}^n( \Vert \cdot \Vert _{{\mathcal {H}}^{-m}} > M)$ for each of the terms on the right-hand side of (4.11).

Let *Y* be a random variable with values in ${\mathcal {H}}^{-m}$. We have that

$$\begin{aligned} {\mathbb {P}}_{\mu _{\textrm{ss}}^n}^n(\Vert Y\Vert _{{\mathcal {H}}^{-m}} > M) \le M^{-2} {\mathbb {E}}_{\mu _{\textrm{ss}}^n}^n[ \Vert Y\Vert ^2_{{\mathcal {H}}^{-m}}]. \end{aligned}$$

Observe that

$$\begin{aligned} |\hat{Y}(k)| \le |Y(\cos (2 \pi k x))| + | Y(\sin (2 \pi kx))|. \end{aligned}$$

Therefore,

$$\begin{aligned} {\mathbb {E}}_{\mu _{\textrm{ss}}^n}^n[ \Vert Y\Vert _{{\mathcal {H}}^{-m}}^2] \le {2}\sum _{k \in {\mathbb {Z}}^d} (1+\Vert k\Vert ^2)^{-m} \big (Y(\cos (2 \pi k x))^2 + Y(\sin (2 \pi kx))^2 \big ). \end{aligned}$$

The relation

$$\begin{aligned} f \mapsto X_0^n(P_T f) \end{aligned}$$

defines by duality a random variable in ${\mathcal {H}}^{-m}$, which for simplicity we just denote by $X_0^n(g_{0,T})$. Using (4.12), we see that

$$\begin{aligned} {\mathbb {E}}_{\mu _{\textrm{ss}}^n}^n[\Vert X_0^n(g_{0,T})\Vert _{{\mathcal {H}}^{-m}}^2]&\le C g_d(n) n^{d-2} e^{2 F'({\rho _*}) T} \sum _{k \in {\mathbb {Z}}^d} (1+\Vert k\Vert ^2)^{-m}\\&\le C g_d(n) n^{d-2} e^{2 F'({\rho _*}) T} \end{aligned}$$

for every $m > d/2$. Recall that our aim is to provide a uniform bound for ${\mathbb {E}}_{\mu _{\textrm{ss}}^n}^n[\Vert X_0^n(g_{0,T})\Vert _{{\mathcal {H}}^{-m}}^2]$. This is the case if we choose

4.18 $$\begin{aligned} T = T_n := \frac{\log ( g_d(n) n^{d-2})}{-2 F'({\rho _*})}. \end{aligned}$$

Now let us estimate the second moment in ${\mathcal {H}}^{-m}$ of the process

4.19 $$\begin{aligned} f \mapsto \int _0^T X_t^n((\partial _t+{\mathbb {L}}_n) g_{t,T}) dt. \end{aligned}$$

Observe that for every $k, k' \in {\mathbb {Z}}^d$,

$$\begin{aligned} \sin (2\pi (k+nk')x) = \sin (2 \pi kx), \quad \cos (2\pi (k+nk')x) = \cos (2 \pi kx). \end{aligned}$$

Therefore, $\hat{X}^n(k+nk') = \hat{X}^n(k)$. The same identity holds for all the processes appearing in (4.11) and in particular for the process defined in (4.19). Take $k \in {\mathbb {Z}}^d$ such that $\Vert k\Vert \le n$. For $f(x) = \sin (2 \pi k x)$ or $f = \cos (2 \pi k x)$, (4.13) gives the bound

$$\begin{aligned} {\mathbb {E}}_{{\mu _{\textrm{ss}}^n}}^n \Big [ \Big | \int _0^T X_t^n ((\partial _t+{\mathbb {L}}_n) g_{t,T}) dt \Big |^2\Big ] \le C g_d(n) n^{d-6} \Vert k\Vert ^8. \end{aligned}$$

For $k \in {\mathbb {N}}$ with $\Vert k\Vert >n$ there exists $k' \in {\mathbb {Z}}^d$ such that $\Vert k -n k' \Vert \le n$. Therefore, (4.13) gives us the bound

$$\begin{aligned} {\mathbb {E}}_{{\mu _{\textrm{ss}}^n}}^n \Big [ \Big | \int _0^T X_t^n ((\partial _t+{\mathbb {L}}_n) g_{t,T}) dt \Big |^2\Big ] \le C g_d(n) n^{d-6} n^8 \le C g_d(n) n^{d+2}. \end{aligned}$$

We conclude that

4.20 $$\begin{aligned} {\mathbb {E}}_{{\mu _{\textrm{ss}}^n}}^n \Big [ \Big \Vert \int _0^T X_t^n((\partial _t+{\mathbb {L}}_n) g_{t,T}) dt \Big \Vert ^2_{{\mathcal {H}}^{-m}}\Big ]&\le C g_d(n)n^{d-6} \Big ( \sum _{\Vert k\Vert \le n} \frac{\Vert k\Vert ^8}{(1+\Vert k\Vert ^2)^{m}}\nonumber \\&\quad + \sum _{\Vert k\Vert > n} \frac{n^8}{(1+\Vert k\Vert ^2)^{m}}\Big )\nonumber \\&\le C g_d(n) n^{2d+2 -2m}, \end{aligned}$$

whenever $m > d/2$, so that the sum $\sum _{\Vert k|>n} (1+\Vert k\Vert ^2)^{m}$ is finite. In particular, (4.20) is bounded in *n* for $m > 3$ if $d=2$ and for $m \ge 4$ if $d=3$. Observe that the bound is independent of *T*, and in particular it holds for $T=T_n$ as defined in (4.18).

Now we will prove tightness of ${\mathcal {M}}_{T_n,T_n}^n(f)$ for $T_n$ chosen as in (4.18). By (4.14), we have that

$$\begin{aligned} {\mathbb {E}}_{{\mu _{\textrm{ss}}^n}}^n\big [ \big \Vert {\mathcal {M}}_{T,T}^n \big \Vert _{{\mathcal {H}}^{-m}}^2\big ] \le C \sum _{k \in {\mathbb {Z}}^d} (1+\Vert k\Vert ^2)^{-m}k^2 \le C \end{aligned}$$

if $m > 1+ d/2$. Since the constant *C* does not depend on *T*, tightness is proved. Observe that the cut-off introduced in (4.20) does not improve the value of *m*.

We are only left to show tightness of the integral term $I_T^n(V)$. Since we only have $L^1({\mathbb {P}}_{{\mu _{\textrm{ss}}^n}}^n)$ bounds for $I_T^n(V; f)$, we need to estimate ${\mathbb {P}}_{\mu _{\textrm{ss}}^n}^n(\Vert I_T^n(V)\Vert _{{\mathcal {H}}^{-m}} > M)$ in a different way. Let $(p(k); k \in {\mathbb {Z}}^d)$ be a probability measure to be chosen in a few lines. We have that

$$\begin{aligned} {\mathbb {P}}_{\mu _{\textrm{ss}}^n}^n(\Vert I_T^n(V)\Vert _{{\mathcal {H}}^{-m}} \ge M )&= {\mathbb {P}}_{\mu _{\textrm{ss}}^n}^n(\Vert I_T^n(V)\Vert _{{\mathcal {H}}^{-m}}^2 \ge M^2 ) \\&= {\mathbb {P}}_{\mu _{\textrm{ss}}^n}^n \Big ( \sum _{k \in {\mathbb {Z}}^d} |\widehat{I_T^n(V)} (k)|^2 (1+ \Vert k\Vert ^2)^{-m} \ge \sum _{k \in {\mathbb {Z}}^d} p(k) M^2\Big ) \\&\le \sum _{k \in {\mathbb {Z}}^d} {\mathbb {P}}_{\mu _{\textrm{ss}}^n}^n \big ( |\widehat{I_T^n(V)}(k)|^2 (1+\Vert k\Vert ^2)^{-m} \ge p(k) M^2 \big )\\&\le \sum _{k \in {\mathbb {Z}}^d} \frac{(1+\Vert k\Vert ^2)^{-m/2} {\mathbb {E}}_{{\mu _{\textrm{ss}}^n}}^n [|\widehat{I_T^n(V)}(k)|]}{p(k)^{1/2}M}. \end{aligned}$$

Using (4.17) we obtain the bound

$$\begin{aligned} {\mathbb {P}}_{\mu _{\textrm{ss}}^n}^n \big (\Vert I_T^n(V)\Vert _{{\mathcal {H}}^{-m}} \ge M \big ) \le C g_d(n) n^{d/2-2}(1+T)\sum _{k \in {\mathbb {Z}}^d} \frac{(1+\Vert k\Vert ^2)^{-m/2}}{M p(k)^{1/2}}. \end{aligned}$$

Choose $p(k) = c(1+\Vert k\Vert ^2)^{-d/2+\delta }$ for $(d-1)/2>\delta >0$, where *c* is the corresponding normalization constant. We obtain the bound

$$\begin{aligned} {\mathbb {P}}_{\mu _{\textrm{ss}}^n}^n(\Vert I_T^n(V)\Vert _{{\mathcal {H}}^{-m}} \ge M )&\le C g_d(n) n^{d/2-2}(1+T) \sum _{k \in {\mathbb {T}}^d} (1+\Vert k\Vert ^2)^{-m/2+d/2+\delta } \\&\le C g_d(n) n^{d/2-2}(1+T) \end{aligned}$$

for every $m> 3d/2$. We conclude that

$$\begin{aligned} {\limsup _{n \rightarrow \infty }}\, {\mathbb {P}}_{\mu _{\textrm{ss}}^n}^n (\Vert I_{T_n}(W)\Vert _{{\mathcal {H}}^{-m}} \ge M ) \le \lim _{n \rightarrow \infty } C g_d(n) n^{d/2-2} \Big (1+ \frac{\log ( g_d(n) n^{d-2})}{-2 F'({\rho _*})}\Big )=0, \end{aligned}$$

from where $I_{T_n}(W)$ converges to 0 in probability with respect to $L^1({\mathbb {P}}_{{\mu _{\textrm{ss}}^n}}^n)$. Since convergence in probability implies tightness, $I_{T_n}(W)$ is tight in ${\mathcal {H}}^{-m}$ for $m >3d/2$. We conclude that $(X^n; n \in {\mathbb {N}})$ is tight in ${\mathcal {H}}^{-m}$ for $m > 3$ in $d=2$ and $m > 9/2$ in $d =3$.

### Convergence

In this section we will complete the proof of Theorem 2.7 by proving the convergence of the finite-dimensional distributions of $(X^n; n \in {\mathbb {N}})$ to $X_\infty$.

Putting estimates (4.12), (4.13) and (4.17) into (4.11), we see that

$$\begin{aligned} {\mathbb {E}}^n_{{\mu _{\textrm{ss}}^n}} \big [ \big | X_{T_n}^n(f) -{\mathcal {M}}_{T_n,T_n}^n(f) \big | \big ]&\le Cg_d(n)^{1/2} n^{d/2-1} \Vert f\Vert _\infty e^{2 F'({\rho _*}) T_n} \\&\quad +C g_d(n)^{1/2} n^{d/2-3} \big ( \Vert D^2 f\Vert _\infty + \Vert D^4 f\Vert _\infty \big )\\&\quad \quad + C \Vert f\Vert _\infty g_d(n) n^{d/2-2}(1+T_n). \end{aligned}$$

For the choice of $T_n$ made in (4.18), we see that

$$\begin{aligned} \lim _{n \rightarrow \infty } \big ( X_{T_n}^n(f) -{\mathcal {M}}_{T_n,T_n}^n(f) \big ) =0. \end{aligned}$$

Therefore, we have reduced the problem of convergence of $(X^n; n \in {\mathbb {N}})$ to the problem of convergence of $( {\mathcal {M}}_{T_n,T_n}^n; n \in {\mathbb {N}})$. We have already proved that the sequence $({\mathcal {M}}_{T_n,T_n}^n; n \in {\mathbb {N}})$ is tight. Therefore, we only need to obtain the limits of the sequences $({\mathcal {M}}_{T_n,T_n}^n(f); n \in {\mathbb {N}})$ for every test function *f*. We will use the following martingale convergence theorem:

#### Proposition 4.4

Let $(M_t^n; t \in [0,T])_{n \in {\mathbb {N}}}$ be a sequence of ${\mathcal {L}}^2$-martingales with càdlàlg trajectories. Let

$$\begin{aligned} \Delta _t^n:= \sup _{0 \le t \le T} | M_{t}^n-M_{t-}^n| \end{aligned}$$

be the size of the largest jump of $(M_t^n; t \in [0,T])$ on the interval [0, *T*] and let $\Phi :[0,T] \rightarrow [0,\infty )$ be a continuous increasing function. Assume that

i) as $n \rightarrow \infty$, $\displaystyle {| \Delta _t^n| \rightarrow 0}$ in probability,
ii) as $n \rightarrow \infty$, $\displaystyle {\langle M^n\rangle _t \rightarrow \Phi (t)}$ in probability for every $t \in [0,T]$.

Under these conditions,

$$\begin{aligned} M_t^n \rightarrow M_t \end{aligned}$$

in law with respect to the $J_1$-Skorohod topology of ${\mathcal {D}}([0,T], {\mathbb {R}})$, where $(M_t; t \ge 0)$ is a continuous martingale of predictable quadratic variation $\langle M\rangle _t = \Phi (t)$ for every $t \in [0,T]$.

This proposition is a restatement of Theorem VIII.3.11 of [15]. Observe that in this proposition, the time window [0, *T*] is fixed, while in our situation $T_n$ grows with *n*. We will overcome this difficulty in the following way. Let $T >0$ be fixed and assume without loss of generality that $T_n \ge T$. Define $(M_t^n; t \in [0,T])$ as

$$\begin{aligned} M_t^n:= {\mathcal {M}}_{T_n-T+t,T_n}^n(f) \end{aligned}$$

for every $t \in [0,T]$. Observe that $(M_t^n; t \in [0,T])$ is a martingale. Its quadratic variation is equal to

$$\begin{aligned} \langle M^n\rangle _t = \int _0^t {\Gamma _n (X^n_{T_n-T+s} (g_{T_n-T+s,T_n}))} ds. \end{aligned}$$

Recall (4.10) and recall the definition of $G(\rho )$ given in (2.5). For every $g: {\mathbb {T}}_n^d \rightarrow {\mathbb {R}}$, let us define

$$\begin{aligned} \varphi _n(g):= \frac{2\chi ({\rho _*})}{n^d} {\!\!\sum _{\begin{array}{c} x, y \in {{\mathbb {T}}^{d}_{n}}\\ x \sim y \end{array}}\!\!}n^2 \big ( g(y)-g(x)\big )^2 + \frac{G({\rho _*})}{n^d} {\sum _{x \in {{\mathbb {T}}^{d}_{n}}}}g(x)^2. \end{aligned}$$

By Theorem 3.4, we have that

$$\begin{aligned}{} & {} {\mathbb {E}}^n_{{\mu _{\textrm{ss}}^n}} \big [ \big ( \Gamma _n X_{T_n\!-T+s}^n(g_{T_n\!-T+s,T_n\!}) - \varphi _n(g_{T_n\!-T+s,T_n\!}) \big )^2 \big ] \\{} & {} \quad \le \frac{Cg_d(n)( \Vert g_{T_n\!-T+s,T_n\!}\Vert _\infty ^2\! +\! \Vert \nabla g_{T_n\!-T+s,T_n\!}\Vert _\infty ^2)}{n^2}. \end{aligned}$$

For simplicity we have estimated the $\ell _n^2$ bounds of the functions appearing in (4.10) by their $\ell ^\infty$ bounds; we are only missing a multiplicative constant that does not affect the computations. Observe that

$$\begin{aligned} \lim _{n \rightarrow \infty } \varphi _n(g_{T_n-T+s,T_n}) =: \widetilde{\varphi }_{T-s}(f) = 2 \chi ({\rho _*})\! \int \!\Vert \nabla P_{T-s} f(x)\Vert ^2 dx + G({\rho _*}) \!\int \! P_{T-s} f(x)^2 dx. \end{aligned}$$

By Taylor’s formula, the trapezoidal rule and the fact that

$$\begin{aligned} D^2 (\nabla P_{T-s}f)^2=2(D^2 P_{T-s}f)^2+ 2 DP_{T-s}f D^3 P_{T-s} f \end{aligned}$$

we get

$$\begin{aligned}&\Big | \frac{1}{n^d} {\!\!\sum _{\begin{array}{c} x, y \in {{\mathbb {T}}^{d}_{n}}\\ x \sim y \end{array}}\!\!}n^2 \big (g_{T_n-T+s,T_n}\big (\tfrac{y}{n}\big ) - g_{T_n-T+s,T_n}\big (\tfrac{x}{n}\big )\big )^2 - \int \Vert \nabla P_{T-s}f(x)\Vert ^2 dx\Big |\\&\quad \le \frac{C}{n^2} \big ( \Vert D P_{T-s}f\Vert _\infty \Vert D^3 P_{T-s}f\Vert _\infty + \Vert D^2 P_{T-s}f\Vert _\infty ^2\big )\\&\quad \le \frac{C}{n^2} \big ( \Vert D f\Vert _\infty \Vert D^3 f\Vert _\infty + \Vert D f\Vert _\infty ^2 \big ) \end{aligned}$$

and

$$\begin{aligned}&\Big |\frac{1}{n^d} {\!\!\sum _{\begin{array}{c} x, y \in {{\mathbb {T}}^{d}_{n}}\\ x \sim y \end{array}}\!\!}g_{T_n-T+s,T_n}\big (\tfrac{x}{n}\big )^2 - \int P_{T-s}f(x)^2 dx \Big |\\&\quad \le \frac{C}{n^2} \big ( \Vert P_{T-s}f\Vert _\infty \Vert D^2P_{T-s}f\Vert _\infty + \Vert D P_{T-s}f \Vert _\infty ^2 \big )\\&\quad \le \frac{C}{n^2} \big ( \Vert f\Vert _\infty \Vert D^2 f\Vert _\infty + \Vert D f \Vert _\infty ^2 \big ). \end{aligned}$$

Since these estimates are uniform in *s*, we conclude that

$$\begin{aligned} \lim _{n \rightarrow \infty } \langle M^n\rangle _t = \int _0^t \widetilde{\varphi }_{T-s}(f) ds \end{aligned}$$

in ${\mathcal {L}}^1({\mathbb {P}}_{{\mu _{\textrm{ss}}^n}}^n)$, and condition ii) of Proposition 4.4 is satisfied for

$$\begin{aligned} \Phi (t) = \Phi _t(f):= \int _0^t \widetilde{\varphi }_{T-s}(f) ds. \end{aligned}$$

The jumps of $(M_t^n; t \in [0,T])_{n\in {\mathbb {N}}}$ at time $t \in [0,T]$ have size at most

$$\begin{aligned} n^{-d/2} \Vert g_{T_n-T+t,T_n}\Vert _\infty \le n^{-d/2} \Vert f\Vert _\infty , \end{aligned}$$

and in particular condition i) of Proposition 4.4 is satisfied. We conclude that $(M_t^n; t \ge 0)_{n\in {\mathbb {N}}}$ converges in law to a continuous martingale of quadratic variation $\{\Phi (t); t \in [0,T]\}$. In particular, the limit in law

$$\begin{aligned} \lim _{n \rightarrow \infty } \big ( {\mathcal {M}}_{T_n,T_n}^n(f) - {\mathcal {M}}_{T_n-T,T_n}^n (f)\big ) = \lim _{n \rightarrow \infty } \big (M_T^n - M_0^n \big ) \end{aligned}$$

exists and it is equal to ${\mathcal {N}}(0,\Phi _T(f))$. Now let us estimate the variance of ${\mathcal {M}}_{T_n-T,T_n}^n(f)$. We have that

$$\begin{aligned} {\mathbb {E}}^n_{{\mu _{\textrm{ss}}^n}} \big [ {\mathcal {M}}_{T_n-T,T_n}(f)^2\big ]&= {\mathbb {E}}^n_{{\mu _{\textrm{ss}}^n}} \big [ \langle {\mathcal {M}}_{T_n-T,T_n}^n(f)\rangle \big ] = \int _0^{T_n-T} {\mathbb {E}}^n_{\mu _{\textrm{ss}}^n}\big [ {\Gamma _n (X_s^n(g_{s,T_n}))}\big ] ds\\&\le \int _0^{T_n-T} \!\!\!\!\! C \big ( \Vert g_{s,T_n}\Vert _\infty ^2 + \Vert \nabla g_{s,T_n} \Vert _\infty ^2 \big ) ds\\&\le \int _0^{T_n-T} C \big ( \Vert f\Vert _\infty ^2 + \Vert \nabla f\Vert _\infty ^2\big ) e^{2 F'({\rho _*}) (T_n-s)} ds\\&\le C \big ( \Vert f\Vert _\infty ^2 + \Vert \nabla f\Vert _\infty ^2\big ) e^{2 F'({\rho _*}) T} . \end{aligned}$$

Since $F'({\rho _*}) <0$, the last line of this estimate converges to 0 as $T \rightarrow \infty$, uniformly in *n*. Therefore, we conclude that

$$\begin{aligned} \lim _{n \rightarrow \infty } {\mathcal {M}}_{T_n,T_n}^n(f) = {\mathcal {N}}(0, \Phi _\infty (f)), \end{aligned}$$

where

$$\begin{aligned} \Phi _\infty (f):= 2 \chi ({\rho _*}) \int _0^\infty \int \Vert \nabla P_t f(x)\Vert ^2 dx dt + G({\rho _*}) \int _0^\infty \int P_tf(x)^2 dx dt. \end{aligned}$$

Observe that $\Phi _\infty (f)$ is also given by (4.5). Now we are ready to complete the proof of Theorem 2.7. By Wald’s device, the law of $X_\infty$ is characterized by the laws of $X_\infty (f)$ for $f \in {\mathcal {C}}^\infty ({\mathbb {T}}^d)$. Let $\widetilde{X}$ be a limit point of $(X^n; n \in {\mathbb {N}})$, which exists by tightness, and let $f \in {\mathcal {C}}^\infty ({\mathbb {T}}^d)$. We have just proved that $\widetilde{X}(f)$ has the same law of $X_\infty (f)$. We conclude that $\widetilde{X} = X_\infty$, and in particular $(X^n; n \in {\mathbb {N}})$ has a unique limit point, which finishes the proof of Theorem 2.7.

## Discussion

We have proved that the CLT fluctuations of the density of particles under the NESS of an example of a driven-diffusive model are given by a Gaussian process presenting non-local correlations. For the sake of clarity, we have presented the proof for one of the simplest driven-diffusive models for which a non-trivial limit appears, that is, for a reaction-diffusion model with quadratic interactions. The restriction to quadratic interactions is not essential; however the restrictions to small parameters $\lambda$ and dimensions $d \le 3$ could not be removed without new arguments.

### General reaction rates

For $\lambda \ge 0$, the reaction rates $c_x$ can be written as $c_x = h_x + \lambda r_x$, where

$$\begin{aligned} h_x(\eta ):= a (1-\eta _x) + b \eta _x \end{aligned}$$

can be interpreted as a chemical reservoir of particles at density $\frac{a}{a+b}$, and

$$\begin{aligned} r_x(\eta ) = \frac{1}{2d} \sum _{y \sim x} \eta _y(1-\eta _x) \end{aligned}$$

can be interpreted as the interaction rate of some chemical reaction. The parameter $\lambda$ corresponds to the intensity of the reaction, and our smallness condition in $\lambda$ corresponds to assuming that the strength of the chemical reaction is uniformly bounded in *n*, with respect to the strength of the chemical bath, by some constant depending only on the density of the bath. For $\lambda \in (-a,0)$ some adjustments are needed, but a similar interpretation is possible. Our results hold for general local reaction rates $r_x$, but always under the boundedness assumption described above.

### MFT and density fluctuations

The correlation operator of the limiting fluctuation density field can be described in terms of thermodynamic functions appearing in MFT. The MFT for reaction-diffusion models like the one presented here has been described in great generality in [22]. One starts from the hydrodynamic equation

$$\begin{aligned} \partial _t \rho = \nabla \cdot \big ( D(\rho ) \nabla \rho \big ) + A(\rho ) - B(\rho ), \end{aligned}$$

where $D(\rho )$ is the diffusivity of the diffusion dynamics, equal to 1 in our case, $A(\rho ):= \int c_x(\eta ) (1-\eta _x) d \nu _\rho ^n$ and $B(\rho ):= \int c_x(\eta ) \eta _x d \nu _\rho ^n$. Then, under the invariant measure ${\mu _{\textrm{ss}}^n}$, the density of particles is concentrated on stable solutions of the elliptic equation

5.1 $$\begin{aligned} \nabla \cdot \big ( D(\rho ) \nabla \rho \big ) + A(\rho ) - B(\rho ) =0. \end{aligned}$$

Observe that in our notation, $F = A-B$ and $G =A+B$. If *F* has a unique zero ${\rho _*}$ on the interval [0, 1] such that $F'({\rho _*}) < 0$, then the elliptic equation (5.1) has a unique solution, which is the constant solution equal to ${\rho _*}$. In [22], large deviations principles were proved for the hydrodynamic and hydrostatic limit of the density of particles of the reaction-diffusion model under the assumption $\lambda \ge 0$. At a formal level, the correlation operator of the CLT for the density of particles corresponds to the Hessian of the rate function at its unique zero. In the case of reaction-diffusion models, such derivation has been accomplished in Section 3.5 of [4]. In our infinite-dimensional context, such expansion is difficult to justify, but *a posteriori* it provides the right answer. In [18] it has been shown that the fluctuations around the hydrodynamic limit follow the stochastic heat equation

SHE $$\begin{aligned} \partial _t X = \nabla \cdot \big ( D({\rho _*}) \nabla X + \sqrt{\chi ({\rho _*})} \dot{{\mathcal {W}}}^1\big ) + F'({\rho _*}) X + \sqrt{G({\rho _*})} \dot{{\mathcal {W}}}^2, \end{aligned}$$

where $\dot{{\mathcal {W}}}^1$ is a space-time white noise with values in ${\mathbb {R}}^d$, $\dot{{\mathcal {W}}}^2$ is a space-time white noise independent of $\dot{{\mathcal {W}}}^1$ and $\chi ({\rho _*}) = {\rho _*}(1-{\rho _*})$ is the *mobility* of the model. The density fluctuations around the hydrostatic limit are given by the stationary solution of this equation. The stationary solution of (SHE) can be obtained from *Duhamel’s formula*:

$$\begin{aligned} X_\infty = \int _0^\infty \sqrt{2\chi ({\rho _*})} \dot{{\mathcal {W}}}^1 \cdot \nabla P_t + \int _0^\infty \sqrt{G({\rho _*})} \dot{{\mathcal {W}}}^2 P_t, \end{aligned}$$

where $P_t:= e^{(D({\rho _*}) \Delta + F'({\rho _*}))t}$. Therefore, $X_\infty$ is a centered, Gaussian process satisfying

$$\begin{aligned} {\mathbb {E}}[X_\infty (f)^2] = \int _0^\infty \big ( \chi ({\rho _*}) \Vert \nabla P_t f\Vert ^2 + G({\rho _*}) \Vert P_t f\Vert ^2\big ) dt. \end{aligned}$$

Observe that

$$\begin{aligned} \int _0^\infty \Vert \nabla P_t f\Vert ^2 dt&= \int _0^\infty \langle P_t f , - \Delta P_t f\rangle dt = \int _0^\infty \big ( -\tfrac{1}{2} \partial _t \langle P_t f ,P_t f\rangle + F'({\rho _*}) \langle P_t f, P_t f\rangle \big ) dt \\&= \tfrac{1}{2} \Vert f\Vert ^2 + F'({\rho _*}) \int _0^\infty \Vert P_t f\Vert ^2 dt. \end{aligned}$$

In particular,

$$\begin{aligned} {\mathbb {E}}[X_\infty (f)^2] = \chi ({\rho _*})\Vert f\Vert ^2 + (G({\rho _*}) + 2 \chi ({\rho _*}) F'({\rho _*}))\int _0^\infty \Vert P_t f\Vert ^2 dt. \end{aligned}$$

One can check that $\lambda$ and $G({\rho _*}) + 2 \chi ({\rho _*}) F'({\rho _*})$ have the same sign. Therefore, the sign of $\lambda$ indicates whether $X_\infty$ has positive or negative correlations. We observe that for $\lambda >0$, one can interpret $X_\infty$ as the sum of a white noise and an independent *massive Gaussian free field*. For $\lambda <0$, it turns out that the sum of $X_\infty$ and an independent massive free field is equal to a white noise.

### Local equilibrium

Since diffusion has a diffusive scaling and reaction does not have scaling, at small scales the effect of the diffusive dynamics is stronger than the effect of the reaction dynamics. Therefore, it is reasonable to expect the reaction-diffusion model to be in *local equilibrium*, meaning that at small scales the law of the density of particles should look like the invariant law of the diffusive dynamics. In our case, this corresponds to a product Bernoulli measure with constant density. In Theorem 2.6, we have proved that this is indeed the case for the NESS, and we have established a mesoscopic scale up to which local equilibrium holds. In dimension $d=1$, we have shown that non-equilibrium behavior only appears at macroscopic scales, since at every mesoscopic scale, the NESS is statistically indistinguishable from a product Bernoulli measure with density ${\rho _*}$. We conjecture that Theorem 2.6 is *sharp*, that is, the scale $R_n$ is the largest scale at which one should expect the NESS to behave as a local equilibrium. Observe that for $d \ge 3$ the covariance operator of the massive free field has a singularity of order $\Vert y-x\Vert ^{2-d}$ at the diagonal ($\log \Vert y-x\Vert$ for $d=2$), which is compatible with the mesoscopic scale $R_n$ stated in Theorem 2.6.

Local equilibrium is one of the axioms from which MFT is derived [5]. Although the validity of MFT has been proved for a range of models, we are not aware of any proof of Theorem 2.6. Therefore, Theorem 2.6 can be seen as the mathematical basis of the derivation of MFT from first principles.

### Absolute continuity of fluctuations with respect to white noise

In this section we prove that the limit fluctuations given by $X_\infty$ are absolutely continuous with respect to a white noise. Recall that our results are for $d\le 3$. Let $(\zeta _{k,i}; k \in {\mathbb {Z}}^d, i =1,2)$ be a sequence of i.i.d. random variables with common law ${\mathcal {N}}(0,1)$ and define $(\xi _k; k \in {\mathbb {Z}}^d)$ as follows. For $k =0$, $\xi _0=\zeta _{0,1}$, and for $k \in {\mathbb {Z}}^d$ with $k_1 >0$,

$$\begin{aligned} \xi _k:= \frac{\zeta _{k,1} + i \zeta _{k,2}}{\sqrt{2}} \text { and } \xi _{-k}:= \frac{\zeta _{k,1} - i \zeta _{k,2}}{\sqrt{2}}. \end{aligned}$$

From (4.5), $X_\infty$ has the representation

$$\begin{aligned} X_\infty (f) = \sum _{k \in {\mathbb {Z}}^d} \hat{f}(k) \xi _{k} \sqrt{\lambda _k}, \end{aligned}$$

where

$$\begin{aligned} \lambda _k:= \frac{4 \pi ^2 k^2\chi ({\rho _*})}{4 \pi ^2 k^2-F'({\rho _*})} + \frac{G({\rho _*})}{8 \pi ^2 k^2 -2 F'({\rho _*})}. \end{aligned}$$

The white noise of variance $\chi ({\rho _*})$ has the representation

$$\begin{aligned} Y(f) = \sum _{k \in {\mathbb {Z}}^d} \hat{f}(k) \xi _{k} \sqrt{\chi ({\rho _*})}. \end{aligned}$$

In other words, in Fourier space, both processes $X_\infty$ and *Y* are represented by tensor products of independent Gaussians. It will be convenient to rewrite the expressions for $X_\infty$ and *Y* as

$$\begin{aligned}{} & {} X_\infty (f):= \hat{f}(0) \zeta _{0,1} \sqrt{\lambda _0} + \sum _{k_1>0} \sqrt{2} \big ( \Re ( \hat{f}(k)) \zeta _{k,1} + \Im (\hat{f}(k)) \zeta _{k,2} \big ) \sqrt{\lambda _k}, \\{} & {} Y(f):= \hat{f}(0) \zeta _{0,1} + \sum _{k_1 >0} \sqrt{2} \big ( \Re ( \hat{f}(k)) \zeta _{k,1} + \Im (\hat{f}(k)) \zeta _{k,2} \big ). \end{aligned}$$

The relative entropy between two centered Gaussians satisfies the formula

$$\begin{aligned} H({\mathcal {N}}(0,\sigma _2^2) | {\mathcal {N}}(0,\sigma _2^1)) =\Xi \Big ( \frac{\sigma _2^2}{\sigma _1^2}\Big ), \end{aligned}$$

where $\Xi (r):= \frac{1}{2} (r - \log r -1)$. In particular, the relative entropy is of order $\frac{1}{4} (\frac{\sigma _2^2}{\sigma _1^2}-1)^2$ as $\frac{\sigma _2^2}{\sigma _1^2} \rightarrow 1$, and therefore for every $M >0$ there exists a finite constant $C_M$ such that

$$\begin{aligned} H({\mathcal {N}}(0,\sigma _2^2) | {\mathcal {N}}(0,\sigma _1^2)) \le C_M \Big (\frac{\sigma _2^2}{\sigma _1^2}-1\Big )^2, \end{aligned}$$

whenever $\frac{\sigma _2}{\sigma _1} \in [\frac{1}{M}, M]$. Recall (4.6). Therefore, there exists $\widetilde{M}$ finite such that

$$\begin{aligned} \frac{\lambda _k}{\chi ({\rho _*})} \in [\widetilde{M}^{-1}, \widetilde{M}] \end{aligned}$$

for every $k \in {\mathbb {Z}}^d$. By the tensorization property of the relative entropy, the relative entropy of the law of $X_\infty$ with respect to the law of *Y* is equal to

$$\begin{aligned} \sum _{k \in {\mathbb {Z}}^d} \Xi \Big (\frac{\lambda _k}{\chi ({\rho _*})}\Big ), \end{aligned}$$

which turns to be bounded by

$$\begin{aligned} \sum _{k \in {\mathbb {Z}}^d} C_{ \widetilde{M}} \Big (\frac{\lambda _k}{\chi ({\rho _*})} -1 \Big )^2. \end{aligned}$$

Observe that there exists a constant *C* such that

$$\begin{aligned} \Big | \frac{\lambda _k}{\chi ({\rho _*})} -1 \Big | \le \frac{C}{1+ \Vert k\Vert ^2}. \end{aligned}$$

Therefore, the sum

$$\begin{aligned} \sum _{k \in {\mathbb {Z}}^d} \Big (\frac{\lambda _k}{\chi ({\rho _*})} -1 \Big )^2 \end{aligned}$$

is finite for $d <4$, from where we conclude that $X_\infty$ has a density with respect to *Y*, as we wanted to show.

From this computation, one could argue that in $d \le 3$, the relative entropy of ${\mu _{\textrm{ss}}^n}$ with respect to $\nu _{{\rho _*}}^n$ should be *uniformly bounded* in *n*. Observe that the entropy estimate of Theorem 2.4 is not uniform for $d=2,3$. Observe that in our methodology, the estimate of the relative entropy of ${\mu _{\textrm{ss}}^n}$ with respect to $\nu _{{\rho _*}}^n$ is obtained as the time integral of the entropy production along the evolution of the process, which in fact transports $\nu _{{\rho _*}}^n$ to ${\mu _{\textrm{ss}}^n}$. There is no *a priori* reason to believe that this particular transport map is near optimal. In [6], the authors introduce a measure $\tilde{\nu }_{{\rho _*}}^n$, which turns out to be a better approximation for the stationary state ${\mu _{\textrm{ss}}^n}$ that $\nu _{{\rho _*}}^n$. We believe that a similar approach could be used in our case to obtain a uniform bound on the relative entropy between ${\mu _{\textrm{ss}}^n}$ and the approximation $\tilde{\nu }_{{\rho _*}}^n$.

### Chemical equilibrium and NESS

It is usual to introduce NESS as invariant states on which there is a *mass flow* (or particle flow) due to asymmetric motion or different boundary conditions. The measure ${\mu _{\textrm{ss}}^n}$ can be understood as a non-equilibrium stationary state in a different way. Let us consider a solution of an ionic salt, like $\textrm{NaCl}$ (sodium chlorine). The chemical equilibrium of this solution is given by

$$\begin{aligned} \textrm{NaCl} \rightleftharpoons \textrm{Na}^+ + \textrm{Cl}^-, \end{aligned}$$

and the corresponding proportions of each state can be computed in terms of the corresponding chemical potential. If we interpret the neutral state as 0 and the ionized state as 1, we see that this chemical equilibrium corresponds to the case $\lambda =0$. In our case, $\lambda > 0$ corresponds to a situation in which the presence of other ions induces ionization, and $\lambda <0$ represents the case in which the presence of other ions prevents ionization. This second interpretation is a bit more realistic, but for our purposes, what matters is that the pair interaction modeled by the parameter $\lambda$ induces a non-reversible stationary state from the point of view of chemical potentials. However, observe that the law of the limiting process $X_\infty$ is an *equilibrium state* of Eq. (4.2). In other words, reversibility is recovered at the scaling limit. This is only true due to the additional dissipation given by the diffusion term of the equation. For general NESS, reversibility is not recovered in the limit. Since we have aimed to describe our methodology in the simplest non-trivial possible case, we ended up with a model for which reversibility is recovered in the limit. The interested reader could check that our methodology allows us to combine our reaction dynamics with a weakly asymmetric system (WASEP) to obtain CLT for a NESS that stays non-reversible in the limit.

## Appendix A: Lemmas

The following estimate is known as *Hoeffding’s lemma*:

### Lemma A.1

Let *X* be a random variable such that $a\le X \le b$ a.s. For every $\theta \in {\mathbb {R}}$,

$$\begin{aligned} \log {\mathbb {E}}[e^{\theta (X - {\mathbb {E}}[X])}] \le \tfrac{1}{8}(b-a)^2 \theta ^2. \end{aligned}$$

The following lemma states that random variables satisfying the bound in Hoeffding’s lemma are subgaussian:

### Lemma A.2

Let *X* be a real-valued random variable such that $\log {\mathbb {E}}[e^{\theta X}] \le \frac{1}{2}\sigma ^2\theta ^2$ for every $\theta \in {\mathbb {R}}$. We have that

$$\begin{aligned} {\mathbb {E}} [e^{\gamma X^2}] \le \frac{1}{\sqrt{1- 2\sigma ^2 \gamma }} \end{aligned}$$

for every $$\gamma < \frac{1}{2\sigma ^2}$$.

### Proof

Let *Z* be a Gaussian random variable of mean zero and unit variance, independent of *X*. We have that taking $\theta = \sqrt{2 \gamma } Z$,

$$\begin{aligned} {\mathbb {E}}[e^{\gamma X^2}] = {\mathbb {E}}[ {\mathbb {E}}[e^{\sqrt{2 \gamma } X Z}|X]] = {\mathbb {E}}[ {\mathbb {E}} [ e^{\sqrt{2 \gamma } X Z} | Z]] \le {\mathbb {E}}[e^{\sigma ^2 \gamma Z^2}] = \frac{1}{\sqrt{1-2\sigma ^2 \gamma }}. \end{aligned}$$

$$\square $$

## Footnotes

1 Here and below, $$\displaystyle {{\!\!\sum _{\begin{array}{c} x, y \in {{\mathbb {T}}^{d}_{n}}\\ x \sim y \end{array}}\!\!}}$$ indicates a sum over *unordered* pairs $x,y \in {{\mathbb {T}}^{d}_{n}}$.
